# Effect of Aqueous Enzymatic Extraction of Deer Oil on Its Components and Its Protective Effect on Gastric Mucosa Injury

**DOI:** 10.3389/fnut.2021.769463

**Published:** 2021-11-16

**Authors:** Yun-Shi Xia, Yin-shi Sun, Chang Liu, Zhi-Man Li, Duo-Duo Ren, Rui Mu, Yan-Ting Zhang, Pan-Pan Bo, Li-juan Zhao, Zi Wang

**Affiliations:** ^1^College of Chinese Medicinal Materials, Jilin Agricultural University, Changchun, China; ^2^Institute of Special Wild Economic Animals and Plants, Chinese Academy of Agricultural Sciences, Changchun, China; ^3^College of Food Science and Engineering, Changchun University, Changchun, China

**Keywords:** deer oil, aqueous enzymatic extraction, ethanol, gastric mucosal injury, MAPK pathway, NF-κB pathway

## Abstract

In this study, deer suet fat was used as a raw material to study the effects of aqueous enzymatic extraction of deer oil on its components, followed by studies into the potential protective activity, and related molecular mechanisms of deer oil on ethanol-induced acute gastric mucosal injury in rats. The results show that aqueous enzymatic extraction of deer oil not only has a high extraction yield and has a small effect on the content of active ingredients. Deer oil can reduce total stomach injury. Without affecting the blood lipid level, it can reduce the oxidative stress, which is manifested by reducing the content of myeloperoxidase (MPO) and enhancing the activity level of superoxide dismutase (SOD) and glutathione peroxidase (GSH-Px). It also enhances the expression of defense factors prostaglandin (E2), epidermal growth factor (EGF), and somatostatin (SS), it inhibits apoptosis evidenced by the enhanced of Bcl-2 and decreased expression of cleavage of caspase-3 and Bax. At the same time, it reduces inflammation, which is manifested by reducing the expression of IL-1β, interleukin 6 (IL-6), and tumor necrosis factor alpha (TNF-α) gastric tissue pro-inflammatory cytokines, and enhancing the expression of anti-inflammatory factors IL-4 and IL-10, and inhibiting the mitogen-activated protein kinase/nuclear factor kappa B (MAPK/NF-κB) signaling pathway in gastric tissue.

## Introduction

A gastric ulcer is a peptic ulcer that occurs in the corners of the stomach, gastric antrum, cardia, and hiatal hernia (1). It is one of the most common diseases worldwide. There are many factors that cause gastric ulcers, such as *Helicobacter pylori* infection, drugs, dietary factors, stress, and abnormal gastric motility, which are more common in middle-aged and elderly people due to chronic exposure to risk factors irregular eating, mental stress, etc. (2). Severe gastric ulcers have complications such as upper gastrointestinal bleeding, ulcer perforation, pyloric obstruction, and even cancer (3).

The most intuitive manifestation of gastric ulcers is gastric mucosal damage. Alcoholism is one of the most important inducing factors. The direct contact of ethanol and mucosa can induce many metabolic and functional changes leading to mucosal damage, with symptoms such as acute gastrointestinal bleeding and diarrhea. It can also cause gastric mucosal necrosis due to acute hemorrhagic lesions, mucosal edema, epithelial dissection, and inflammatory cell infiltration. The pathogenesis of ethanol-triggered gastric ulcers is multifactorial and includes mucosal inflammation, oxidative stress, and epithelial cell apoptosis. The influx of activated neutrophils into gastric tissues triggers the release of diverse proinflammatory cytokines that amplify the inflammatory process in gastric mucosa (4). In the context of mucosal inflammation, the nuclear factor kappa B (NF-κB) pathway is considered a core participant in the inflammatory environment. Driven by proinflammatory cytokines and reactive oxygen species (ROS), activated NF-κB increases. The transcription of various inflammatory signals can enhance the occurrence of inflammation. In addition, the mitogen-activated protein kinase (MAPK) pathway involves p38MAPK, extracellular signal-regulated kinase (ERK) and cJun N-terminal kinase (JNK), which are transduction pathways that express various protoinflammatory mediators and apoptosis signals (5). Currently, the main clinical treatment of gastric ulcers includes the use of synthetic anti-secretory drugs, such as H2 receptor antagonists (cimetidine) or proton pump inhibitors (omeprazole), antibiotics (clarithromycin), and antacids. Although these synthetic drugs have high purity, their long-term use may also cause a variety of side effects (6). Therefore, it is necessary to find safe and effective drugs from nature (7).

Deer oil is the fatty oil of the sika deer (*Cervus nippon*) or red deer (*Cervus elaphus*). It is also called deer fat. It is a widely sourced natural biological resource with easy access. The extraction of deer oil mainly adopts the boiling method, and the aqueous enzymatic method of extracting oil has mild conditions, and the enzyme-related degradation products do not react with the oil, which can effectively protect the oil, protein, and secondary degradation products, etc. The obtained oil has high purity and good quality (8). Current research into the composition of deer oil has focused on fatty acids and amino acids, which are easily absorbed by the skin to prevent water loss and delay aging. Deer oil has a certain antioxidant activity, so it has a certain development prospect in cosmetics and health products industry. According to research of lard and other animal fats, deer oil has a significant protective effect on acute gastric mucosal injury caused by ethanol (9). However, the protective mechanism of deer oil on gastric mucosal damage is still not understood. Based on the known protective effect of deer oil against gastric mucosal injury, the protective effect of deer oil form on alcohol-induced gastric mucosal injury and its possible mechanism were explored.

## Materials and Methods

### Materials and Animals

Animals and reagents were procured as follows: 8-week-old SD rats, weighing 170–180 g, were purchased from Liaoning Changsheng Biotechnology Co., Ltd., certificate number SCXK (Liao) 2015-0001; deer oil: unprocessed fresh suet fat from Changchun Shilu Deer Industry Co., Ltd.; neutral protease and alkaline protease: Solarbio; superoxide dismutase (SOD) and glutathione peroxidase (GSH-Px) kits: Nanjing Jiancheng Institute of Biological Engineering; myeloperoxidase (MPO), rat tumor necrosis factor alpha (TNF-α), rat interleukin 6 (IL-6), rat interleukin 4 (IL-4), rat interleukin 10 (IL-10), prostaglandin (E2), epidermal growth factor (EGF), and somatostatin (SS) kits: Shanghai Enzyme-Linked Biotechnology Co., Ltd. (MLBIO); anhydrous ethanol: Sinopharm Chemical Reagent Co., Ltd.; physiological saline: Kunming Nanjiang Pharmaceutical Co., Ltd.; sodium pentobarbital and 10% neutral formalin: Beijing Coolaibo Technology Co., Ltd.; IκBα(H-4), NFκB, p38α/β MAPK(A-12), ERK(D-2), JNK(D-2), p-NFκB p65(27.Ser 536), p-JNK(G-7), p-p38 MAPK(D-8), p-ERK(E-4), p-IκBα, IKKα/β, GAPDH: Santa Cruz Biotechnology, Int; caspase-3, Bcl-2, Bax: abcam.

### Water Extraction of Deer Oil

The deer oil was extracted in the laboratory according to the optimal extraction process determined in literature (10). Accurately weigh 200 g of washed and drained frozen deer suet oil, and use a blender to chop it into small pieces. Under the conditions of solid-liquid ratio 1:1, compound enzyme ratio (neutral protease and alkaline protease) 1:3, compound enzyme dosage 2%, extraction temperature 55°C, extraction time 2.5 h, pH 7.0. Then use the vacuum drying method to remove excess water, deer oil was obtained.

### Determination of Basic Indicators

#### Determination of the Yield

Record the weight of the initial fresh deer suet oil before extraction and the quality of the deer oil extracted after filtration (11).

Yield = The quality of filtered deer oil/Weight of initial fresh Luer Suet before extraction × 100%.

#### Moisture Determination

Determined according to China GB5009.3-2016.

### Composition Determination

#### Protein

Weigh 2–5 g of sample (approximately equivalent to 30–40 mg nitrogen), accurate to 0.001 g, detected on a fast nitrogen analyzer. The CR temperature is above 1,030°C, and the RR temperature is above 650°C.

#### Phospholipids

Refer to the molybdenum blue colorimetric method in the China standard SN/T 3851-2014.

#### Cholesterol

Weigh 250 mg deer oil (accurate to 1 mg) into a 25 ml flask, add 1.0 ml betulin internal standard solution. Add 5 ml of potassium hydroxide-ethanol solution, heat to keep it boiling for 15 min, add 5 ml of ethanol and shake well. Pipette 5 ml of the above liquid into the prepared alumina column, collect the eluent, first eluting with 5 ml of ethanol, then eluting with 30 ml of ether, and remove the solvent in the flask with a rotary evaporator.

Gas chromatographic conditions: Stationary phase: SE-54, length 50 m, inner diameter 0.25 mm, film thickness 0.10 μm; Carrier gas: helium, carrier gas flow rate: 36 cm/s, split ratio 1:20; Detector temperature and inlet temperature: 320°C; The column temperature adopts the programmed heating method, which is increased from 240 to 255°C at a rate of 4°C/min; injection volume: 1 μl.

#### Fatty Acids

##### Methyl Ester Treatment

Weigh 150 mg deer oil, add 8 ml of 2% NaOH-methanol solution, and reflux at 80 ± 1°C until the oil droplets disappear. Cool down to room temperature, add 7 ml of 15% BF_3_-methanol solution, reflux at 80 ± 1°C for 3 min, take it out, and quickly cool to room temperature. Accurately add 15 ml of n-heptane, vortex for 2 min, add saturated NaCl aqueous solution, and stand to separate layers. Pipette about 5 ml of the upper n-heptane solution into a 50 ml centrifuge tube containing 3–5 g of anhydrous sodium sulfate, vortex for 1 min, centrifuge, and dilute the supernatant by 10 times or 100 times for injection.

##### GC/MS Instrument Conditions Chromatographic Column

DB-23 60 m × 0.25 × 0.25 μm; Carrier gas: high purity He; Carrier gas flow: 1.0 ml/min; Inlet: 220°C; EI source: 230°C; Program temperature rise conditions: the initial temperature is 60°C, keep for 1 min, 10°C/min, increase to 180°C, then 3°C/min to 220°C, hold for 2 min.

### Gastric Mucosal Injury Caused by Ethanol

#### Rat Body Weight and Organ Index

Thirty healthy male SD rats were selected and kept in an environment with a temperature of 22–25°C and a relative humidity of 45–65%. They were free to drink and eat. After 1 week of adaptive feeding, they were randomly divided into five groups with six rats in each group: indicated as the normal group, model group, positive control drug (0.1 g/kg cimetidine) group, low-dose deer oil group (0.50 g/kg, L-Deer oil), high-dose deer oil group (0.85 g/kg, H-Deer oil). Weights were recorded for the predose period (before dosing on the 1st day), midterm (before dosing on the 15th day), and final period (before the 30th day of dosing). The normal group and the model group were given normal saline; the deer oil groups were given 10 ml/(kg·bw) once a day for 30 consecutive days. After the last administration, food and water were withheld for 24 h. Except for the normal group, rats in the other groups were given 1.0 ml of absolute ethanol per rat. After 1 h, the rats were anesthetized with sodium pentobarbital, and the heart was sacrificed. The gastric tissue was taken and weighed (12–14).

#### Histopathological Examination of Rat Gastric Mucosa

After blood was collected and the rat was sacrificed, whole gastric tissue was collected, the pylorus was ligated, and 10% formalin was perfused. After fixation for 20 min, the stomach was cut along its greater curvature. The gastric surface contents were washed with normal saline, and the gastric mucosa was unfolded with a Vernier caliper. The length and width of the bleeding zone and the number of bleeding points were recorded, and the score was determined according to the bleeding of the gastric mucosa in Table 1, according to the scoring method in Xia et al. (8). After observation, the most severely injured part of the gastric mucosa was selected, fixed in 10% neutral formalin, washed with water, dehydrated, and immersed in wax, which was sliced continuously at a thickness of 5 μm followed by deparaffinization to water. After H&E staining, pathological changes in rat stomach tissues were observed under a microscope (15, 16).

**Table 1:** Criteria for visual observation of ethanol in acute gastric mucosal injury in rats.

**Scoring**	**1**	**2**	**3**	**4**
Blood point	1			
Length of bleeding zone (mm)	1~5	5~10	10~15	>15
Width of bleeding zone (mm)	1~2	>2	–	–

*Each bleeding point on the gastric mucosa is assigned one point, meaning that a bleeding band with width >2 will be assigned two points*.

### Determination of Cytokines

#### Determination of Blood Biochemical Indicators

The blood of the rat was centrifuged at 2,000 rpm/min for 20 min at 4°C, serum was drawn, and triglyceride (TG), total cholesterol (T-CHO), high-density lipoprotein (HDL-C), and low-density lipoprotein (LDL-C) contents were measured.

#### Analysis of SOD, GSH-Px, and MPO Levels in Gastric Tissue

Approximately 15 mg of gastric tissue was homogenized in physiological saline, centrifuged at 12,000 rpm/min for 10 min at 4°C, and the supernatant was stored at −80°C after collection. The protein content of the homogenate was determined by the BCA protein determination method, and the levels of SOD, GSH-Px, and MPO in the gastric tissue were determined according to the kit-supplied method.

#### Determination of Inflammatory and Defensive Factors in Gastric Tissue

The levels of IL-4, IL-6, IL-10, TNF-α, E2, EGF, and SS were measured according to the manufacturers' protocols, and the absorbance was measured at 450 nm with a microplate reader.

### mRNA Expression in Gastric Tissue

Total RNA in the stomach tissue was extracted with the Easystep Super Total RNA Extraction Kit. After the RNA concentration was detected by a Quick Drop, the integrity was verified by 1% agarose gel electrophoresis. The isolated RNA was reverse transcribed into cDNA according to the BioRT cDNA synthesis kit method, and reverse transcription-PCR (RT-PCR) was performed using the gene-specific primers described in Table 2, with the expression level of GAPDH mRNA as a built-in parameter. Finally, agarose gel electrophoresis containing ethidium bromide was used to observe the PCR-amplified products with visualization using a computer image analysis system for semiquantitative analysis.

**Table 2:** Sequences of primers for RT-PCR amplification.

**Target gene**	**Primer sequence (5′–3′)**	**Length (bp)**
IL-1β	TGCTGATGTACCAGTTGGGG CTCCATGAGCTTTGTACAAG	245
IL-6	GCCCTTCAGGAACAGCTATG CAGAATTGCCATTGCACAAC	240
TNF-α	TGATCGGTCCCAACAAGGA TGCTTGGTGGTTTGCTACGA	140
EPO	ACCACTCCCAACCCTCATCAA CGTCCAGCACCCCGTAAATAG	325
EPOR	TGGATGAATGGTTGCGAC TTTGAAGCCAAGTCAGAG	127
GAPDH	AGGTCGGTGTGAACGGATTTG TGTAGACCATGTAGTTGAGGTCA	123

### Western Blot Analysis

Protein was extracted from gastric homogenates with lysis buffer, and protein concentration was measured using the BCA method. Proteins were separated on a 10–15% SDS-PAGE gel and transferred to membrane at constant current of 200 mA. The membrane was then incubated with milk powder for 2 h, washed with PBST for 30 min, incubated with the primary antibody at 4°C overnight, washed again with PBST, and the corresponding secondary antibody was added and incubated with slow shaking at room temperature for 2 h. After the secondary antibody was washed away, HRP chemiluminescence detection was used for visualization and Image G image analysis. The net gray value of the band was compared with the measurement of the internal reference GAPDH, the ratio was calculated, and the differences between the groups were compared.

### Statistical Analysis

All data are expressed as the means ± standard deviation (SD) and analyzed with GraphPad Prism 5.0. One-way analysis of variance (one-way ANOVA) was used to test the differences between groups, and *P* < 0.05 indicated significant differences.

## Results

### The Influence of Aqueous Enzymatic Extraction of Deer Oil on Basic Indicators

The deer oil extracted by the aqueous enzymatic method is a light yellow transparent liquid, which may be because the low temperature extraction has little effect on the appearance and color of the deer oil. The aqueous enzymatic method uses related enzymes to degrade the oil cells and release the oil from the oil cells. In this experiment, the compound enzymes screened in the laboratory were used to extract deer oil, so that the enzymatic hydrolysis reaction proceeded more completely, so the yield of deer oil was higher. In Table 3, The water content of deer oil extracted by hydroenzymatic method was 4.33 ± 0.55%, the protein content was 0.12 ± 0.01%, the phospholipid content was 0.48 ± 0.01 mg/100 g, and the cholesterol content was 44.40 ± 0.96 mg/100 g. Life Science Identifiers.

**Table 3:** The effect of aqueous enzymatic extraction of deer oil on basic indicators.

**Appearance characteristics**	**Yield (%)**	**Water content (%)**	**Protein (%)**	**Phospholipids (mg/100 g)**	**Cholesterol (mg/100 g)**
Light yellow transparent liquid	85.63 ± 7.39	4.33 ± 0.55	0.12 ± 0.01	0.48 ± 0.01	44.40 ± 0.96

### Influence of Aqueous Enzymatic Extraction of Deer Oil on Fatty Acid

It can be seen from Table 4 that a total of 20 fatty acids were detected in the deer oil extracted by the aqueous enzymatic method. Among them, there are five kinds of fatty acids with content >5%, which are palmitic acid (C16:0), oleic acid (C18:1n9C), stearic acid (C18:0), palmitoleic acid (C16:1n7), and linoleic acid. Acid (C18:2n6). Nine kinds of saturated fatty acids, mainly including palmitic acid, stearic acid, myristic acid, etc., accounting for 57.44% of the total fatty acid; four types of monounsaturated fatty acids, including oleic acid, palmitoleic acid, etc., account for 28.87% of the total fatty acids; seven types of polyunsaturated fatty acids, including linoleic acid, linolenic acid, etc., account for 6.00% of the total fatty acids.

**Table 4:** Fatty acid content of deer oil extracted by aqueous enzymatic method.

**Category**	**Fatty acid composition**	**Absolute content (g/100 g)**
	C10:0	0.02
	C12:0	0.15
	C13:0	0.06
	C14:0	2.79
Saturated Fat Acid	C15:0	0.81
(SFA)	C16:0	33.90
	C17:0	0.82
	C18:0	18.80
	C20:0	0.09
	Total	57.44
	C14:1n5	0.61
	C16:1n7	6.28
Monounsaturated fatty acids	C18:1n9C	21.70
(MUFA)	C20:1	0.28
	Total	28.87
	C15:1n5	0.01
	C18:2n6	5.55
	C18:3n3	0.23
Polyunsaturated fatty acids	C20:2n6	0.08
(PUMA)	C20:3n6	0.04
	C20:3n3	0.01
	C20:4n6	0.08
	Total	6.00
Total fatty acid(TFA)	Total	92.31

### The Effect of Deer Oil on Gastric Mucosal Injury Induced by Ethanol in Rats

In Table 5, during the test period, the rats in each group continued to increase their body weight, and there was no significant difference compared with the normal group (*P* > 0.05). Compared with the normal group, the organ index of the model group was significantly higher than that of the normal group (*P* < 0.001), with evidence such as bleeding and edema apparent, indicating success of the model; the organ index, gastric mucosal congestion area, and injury score index of rats in each administration group were reduced to a certain extent compared with the model group (*P* < 0.001, *P* < 0.01, *P* < 0.05).

**Table 5:** Effect of deer oil on acute gastric mucosal injury indices in rats (x ± S).

**Group**	**Dose (g/kg)**	**Initial body weight (g)**	**15 days body weight (g)**	**30 days body weight (g)**	**Viscera index**	**Congestion area (mm)**	**Injury integral index**
Normal	0	168.42 ± 7.33	272.81 ± 10.90	319.98 ± 18.34	0.0055	–	–
Model	0	168.37 ± 5.43	287.95 ± 12.18	334.65 ± 18.66	0.0079^###^	75.56 ± 11.40	39.76 ± 3.41
Cimetidine	0.10	167.37 ± 6.21	289.32 ± 9.86	329.64 ± 11.53	0.0061^***^	25.94 ± 4.95^***^	18.13 ± 4.22^*^
L-deer oil	0.50	170.53 ± 6.03	294.97 ± 9.64	371.54 ± 15.07	0.0064^***^	17.11 ± 4.91^***^	13.40 ± 3.65^**^
H-deer oil	0.85	173.53 ± 4.98	299.12 ± 8.64	382.72 ± 10.56	0.0059^***^	13.40 ± 4.16^***^	10.65 ± 5.27^***^

### *P < 0.001 vs. ethanol-induced ulcer model group*,

* *P < 0.05*,

** *P < 0.01*,

*** *P < 0.001 vs. normal control group*.

**Figure 1:**
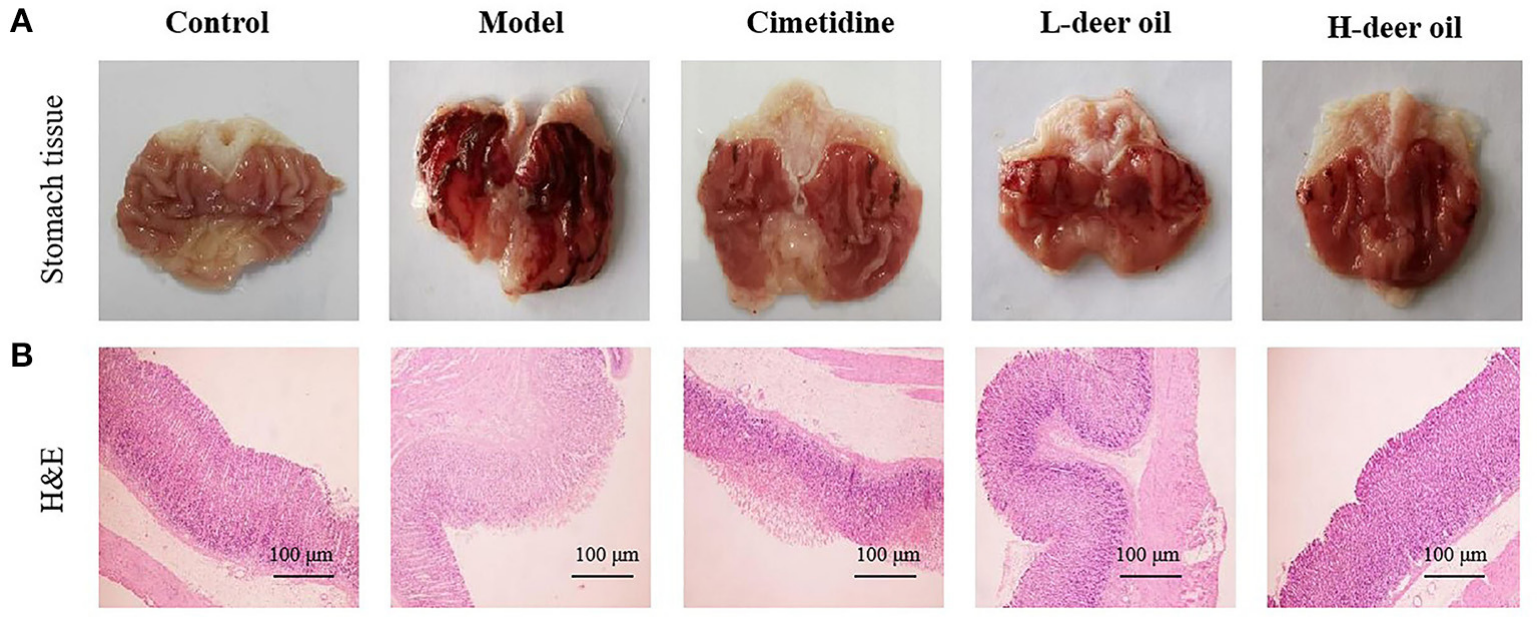
Effects of deer oil on gastric mucosal injury induced by alcohol in rats. **(A)** Representative photos showing macroscopic gastric injury. **(B)** Deer oil alleviate ethanol-induced gastric histopathological damage. Representative photomicrographs of gastric tissues harvested 1 h after ethanol administration (H & E, 40×).

As shown in Figure 1A, the gastric mucosa of rats in the normal group was intact and smooth, without bleeding points or bands; the gastric mucosal surface of rats in the model group was severely congested and swollen, with darker and thicker bleeding bands; the gastric mucosa of rats in the positive drug group had a small amount of slight bleeding on the surface of the gastric mucosa, and the color was lighter; the rats in the deer oil group had slight local bleeding on the gastric mucosa, with small bleeding bands and bleeding spots on the surface.

The results of H&E staining showed that the gastric mucosal cells of the normal group were tightly arranged and ordered, the cells were darker in blue and purple, and the overall gastric mucosal morphology was complete, with clear and obvious boundaries; the gastric mucosal cells of the model group were loosely arranged, disordered, and hollow. Cells aggregated into pieces, with inflammatory infiltration and swelling of the gastric mucosa; the cells of the deer oil group was arranged in an orderly manner, and the fragmented cells were significantly reduced. In addition, the deer oil treatment groups showed dose-dependent protection of gastric mucosal morphology (Figure 1B).

### The Effect of Deer Oil on Cytokines in Rats

**Figure 2:**
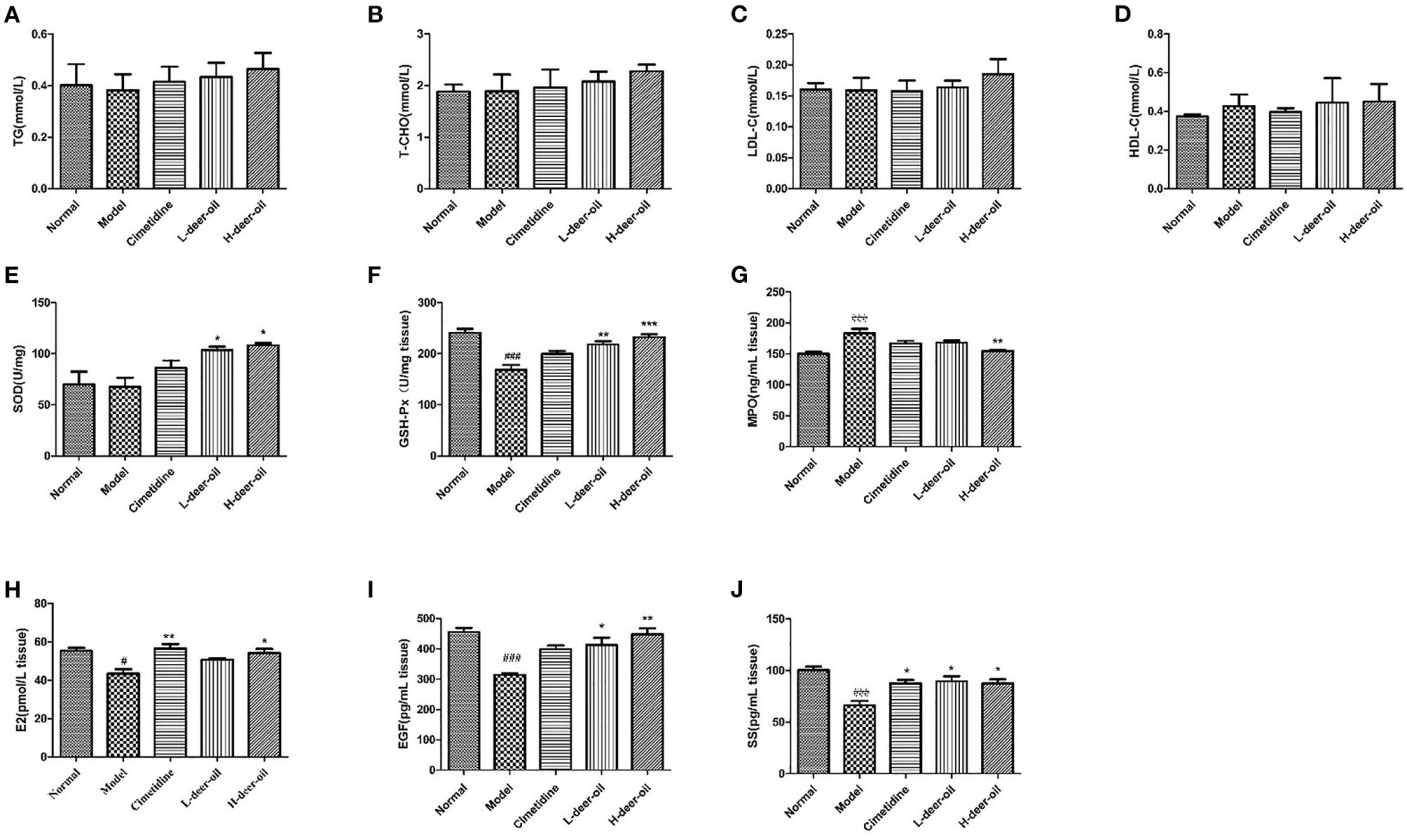
Effects of deer oil on cytokines in rats. **(A)** Gastric TG; **(B)** Gastric T-CHO; **(C)** Gastric LDL-C; **(D)** Gastric HDL-C; **(E)** Gastric SOD; **(F)** Gastric GSH-Px; **(G)** Gastric MPO; **(H)** Gastric E2; **(I)** Gastric EGF; **(J)** Gastric SS. Data are presented as the means ± SD (*n* = 6). ^###^*p* < 0.001 vs. Normal group, ^#^*p* < 0.05 vs. Normal group,***p < 0.001 vs. Model group, **p < 0.01 vs. Model group,*p < 0.05 vs. Model group.

As shown in Figure 2, compared with the normal group, the blood lipid levels of rats in each administration group increased to a certain extent, but there was no significant difference (*P* > 0.05). Compared with the normal group, the GSH-Px activity of the model group decreased (*P* < 0.001), the MPO level increased (*P* < 0.05), and the activity of E2, EGF, and SS decreased (*P* < 0.05, *P* < 0.001), indicating that absolute ethanol caused oxidative stress in rats and a decrease in the level of defensive factors. Compared with the model group, pretreatment in the positive drug group increased the activity of E2 and SS (*P* < 0.05); pretreatment with deer oil effectively increased the activity of SOD and GSH-Px (*P* < 0.01, *P* < 0.001) and reduced the MPO level (*P* < 0.05), increased the activity of EGF and SS (*P* < 0.05, *P* < 0.01), and was dose-dependent.

### The Effect of Deer Oil on Cell Apoptosis and Inflammation

**Figure 3:**
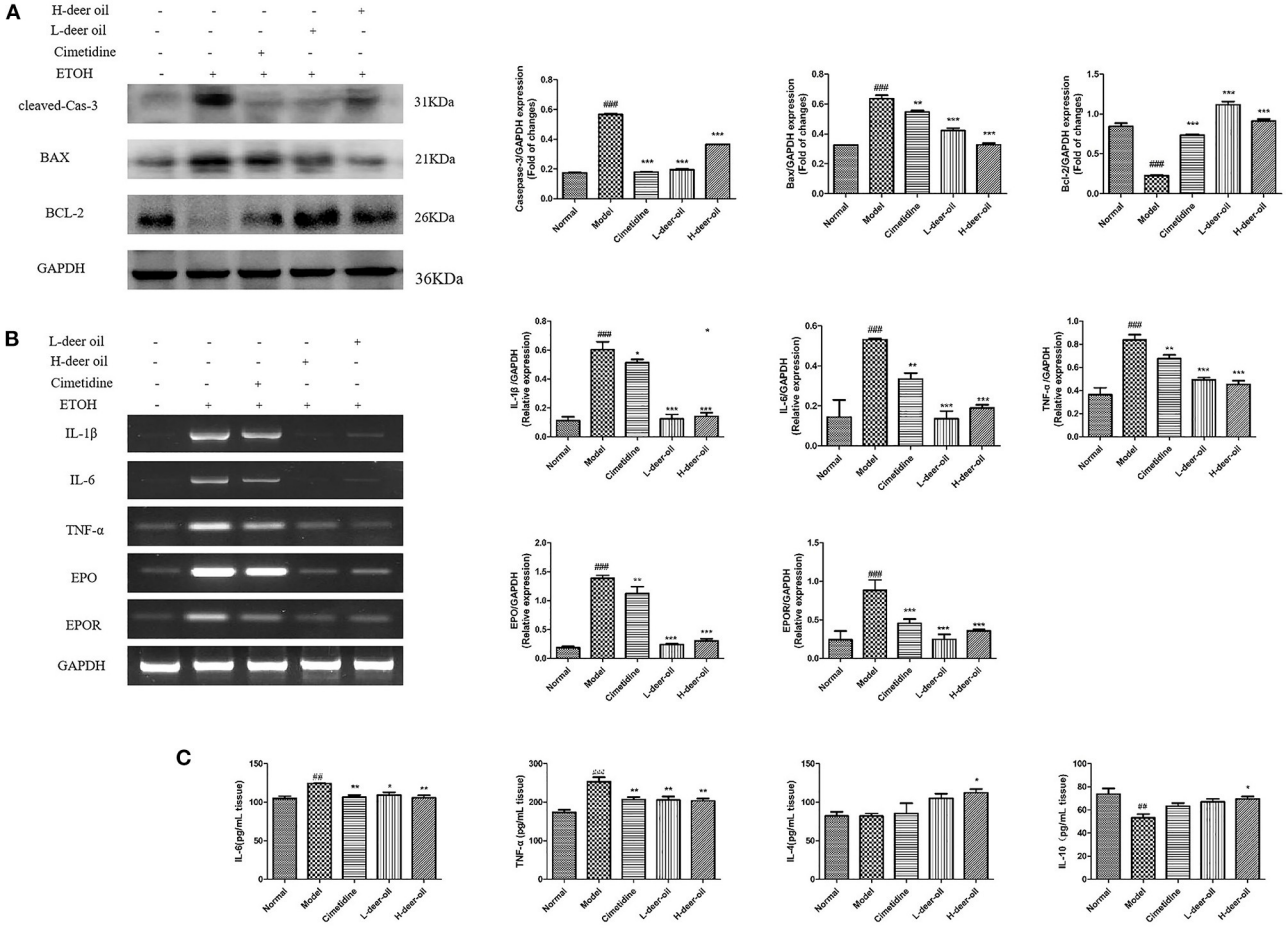
Deer oil weaken the apoptosis and inflammation caused by ethanol in the stomach. **(A)** Western blotting showed the protein expression levels of apoptosis markers. The loading control was GAPDH. **(B)** mRNA expression of inflammatory-related factors and their expression levels. **(C)** Inflammatory factor content in gastric tissue. Data are presented as the means ± SD (*n* = 6). ^###^*p* < 0.001 vs. Normal group, ^##^*p* < 0.01 vs. Normal group, ***p < 0.001 vs. Model group, **p < 0.01 vs. Model group,*p < 0.05 vs. Model group.

As shown in Figure 3A, compared with the normal group, the cleavage of caspase-3 increased (*P* < 0.001), the expression of Bax increased (*P* < 0.001), and the expression of Bcl-2 decreased (*P* < 0.001) in the model group. Ethanol induced apoptosis of gastric mucosal cells. Compared with the model group, deer oil inhibited the protein expression of cleaved caspase-3 and Bax (*P* < 0.001) and increased the expression of Bcl-2 (*P* < 0.001), thereby reducing the occurrence of cell apoptosis. This shows that the protective effect of deer oil on gastric mucosal damage is related to the inhibition of cell apoptosis, and the effect is better than that of the positive drug group.

ELISA kits were used to determine the levels of proinflammatory cytokines and anti-inflammatory cytokines in gastric tissue. As shown in Figure 3C, compared with the normal group, the IL-6 and TNF-α levels of the model group were increased (*P* < 0.05, *P* < 0.001), and the anti-inflammatory cytokines IL-4 and IL-10 were not obviously changed or decreased significantly (*P* < 0.001). Compared with the model group, the deer oil pretreatment groups had decreased proinflammatory cytokine contents and increased anti-inflammatory cytokine contents (*P* < 0.05, *P* < 0.01). Based on the above experimental results, to further evaluate the role of inflammation in ethanol-induced gastric mucosal damage, RT-PCR was performed on the deer oil group to detect the changes in IL-1β, IL-6, TNF-α, EPO, and EPOR mRNA in gastric tissue. As shown in Figure 3B, compared with the normal group of rats, the mRNA expression levels of inflammatory factors EPO and EPOR in the gastric tissue of the model group was significantly increased (*P* < 0.001); the mRNA expression levels of each index in the deer oil were significantly reduced (*P* < 0.001). These data show that after ethanol intake in the model group, a large amount of proinflammatory factors are secreted, leading to the increase of EPO and EPOR levels; the deer oil pretreatment groups secreted less inflammatory factors, the inflammatory factors IL-1β, IL-6, TNF-α, EPO, and EPOR were lower than those of the model group, and the effect was better than that of the positive control drug group.

### Deer Oil Block the Activation of the NF-κB Pathway Induced by Ethanol

**Figure 4:**
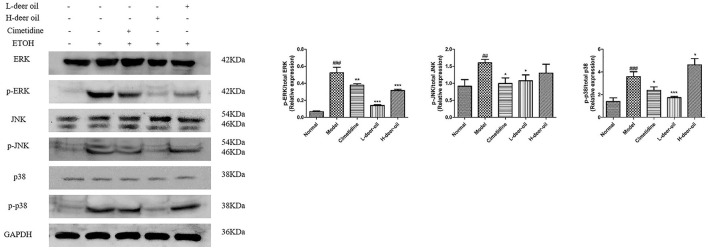
Deer oil blocked ethanol-induced activation of NF-κB pathways. Protein expression levels of the NF-κB signaling pathway were detected by Western blotting. The loading control was GAPDH. Data are presented as the means ± SD (*n* = 6). ^###^*p* < 0.001 vs. the normal group, ^##^*p* < 0.01 vs. the normal group, ***p < 0.001 vs. the model group, **p < 0.01 vs. the model group, *p < 0.05 vs. the model group.

In view of the above-mentioned oxidative stress and inflammation caused by ethanol damage, we clarified the mechanism of deer oil through the NF-κB signaling pathway, which is usually involved in the inflammatory signaling cascade. As shown in Figure 4, compared with the normal group, absolute ethanol stimulation significantly increased the expression of IKK, p-IKBα, and p-NF-κB (*P* < 0.01, *P* < 0.001). Compared with the model group, deer oil significantly reduced the expression of IKK (*P* < 0.01), and the positive control group, deer oil and powdered oil groups all significantly reduced the expression of p-IKBα and p-NF-κB p65 (*P* < 0.01, *P* < 0.001). These results show that deer oil and its powdered oil exert gastric protective effects by blocking the NF-κB signaling pathway, and the effect is better than that of the positive control drug group.

### Deer Oil Block the Activation of the MAPK Pathway Induced by Ethanol

**Figure 5:**
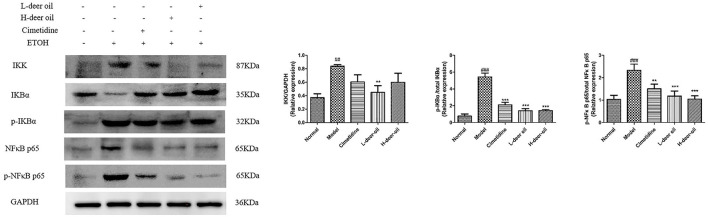
Deer oil blocked ethanol-induced activation of MAPK pathways. Protein expression levels of the MAPK signaling pathway were detected by Western blotting. The loading control was GAPDH. Data are presented as the means ± SD (*n* = 6). ^###^*p* < 0.001 vs. the normal group, ^##^*p* < 0.01 vs. the normal group, ***p < 0.001 vs. the model group, **p < 0.01 vs. the model group vs. the model group.

Ethanol injury also activated three major subgroups in the MAPK family, namely, p-ERK, p-JNK, and p-p38. As shown in Figure 5, compared with the normal group, the expression of p-ERK, p-JNK, and p-p38 in the model group was significantly upregulated (^###^*P* < 0.001). Compared with the model group, the deer oil had reduced expression of p-ERK, p-JNK, and p-p38 (^*^P < 0.05, ^**^P < 0.01, ^***^P < 0.001). These results show that deer oil exert gastric protective effects by blocking the MAPK signaling pathway.

## Discussion

The extraction rate of deer oil extracted by aqueous enzymatic method is higher than that of conventional boiling method (17), reaching 85.63%, but at the same time the water content is also higher, which is 4.33%. The excess water can be removed by direct drying or vacuum drying. The deer oil extracted by this method has a protein content of 0.12%, a phospholipid content of 0.48 mg/100 g, and a cholesterol content of 44.40 mg/100 g. The content of unsaturated fatty acids in deer oil extracted by aqueous enzymatic method reached 34.87%. This may be because the low-temperature extraction method of aqueous enzymatic method had less effect on the content of unsaturated fatty acids (18). In summary, the deer oil extracted by the aqueous enzymatic method is of high quality and lighter in color than the boiled method. It not only has a high extraction rate but also has a small impact on the content of active ingredients. It is a safe and effective green extraction method. The development and utilization of cosmetics and other fields provide a theoretical basis.

Excessive intake of absolute ethanol can cause acute gastric mucosal damage and trigger oxidative stress, apoptosis and inflammatory pathways. The rat gastric tissues swelled and hemorrhaged after being stimulated by absolute ethanol, and the model was successfully established. Compared with the model group, the weight of stomach tissue or body mass in each administration group was significantly reduced, and edema was reduced to a certain extent. Deer oil reduced the acute gastric mucosal injury caused by ethanol, and the effect of the high-dose group was better than that of the low-dose group.

Compared with the normal group, the blood lipid levels of the rats in each administration group increased to a certain extent, but there was no significant difference. This may be due to the small amount of fat given to rats every day, which did not reach the dose that would cause the blood lipid level to rise. Oxidative stress plays a key role in the pathogenesis of gastric mucosal damage. The intake of absolute ethanol will reduce the antioxidant capacity of cells, leading to the occurrence of oxidative stress, which in turn causes the body to produce an inflammatory response (19). Glutathione peroxidase and SOD are important endogenous antioxidant enzymes and the first line of defense against oxidative damage. Superoxide dismutase activity can reflect the body's ability to scavenge free radicals. Glutathione peroxidase can block the free radicals caused by peroxides from causing damage to the body. Myeloperoxidase is a key indicator to assess the degree of neutrophil infiltration into the gastric mucosa (20). In the present study, a significant decrease of SOD activity and GSH level and increase of MDA level were observed in ethanol-induced gastric mucosal injury. However, deer oil reduced MDA level and increased SOD activity and GSH-Px level, which may has a protective effect on gastric mucosa as seen by the reduction of gastric mucosal injury.

Epidermal growth factor is an important protective factor of the gastrointestinal tract and has a strong protective effect on stomach and duodenal injury. It is an important intermediary factor that regulates ulcer healing. Epidermal growth factor can reduce the poor renewal of gastric mucosal cells and weaken defense ability. Ethanol can also inhibit the responsiveness of EGF to gastric mucosal injury (21). Prostaglandin E2 (PGE2) is an important regulatory mediator of the gastric mucosa that changes when the gastric mucosa is damaged for a variety of reasons. It mainly enhances the protective ability of gastric mucosa by promoting gastric mucus production, inhibiting gastric acid secretion, reducing inflammation, improving gastric mucosal blood circulation, and stimulating the production of gastric mucosal protective growth factors (22). The synthesis and secretion of somatostatin SS (growth hormone release inhibitory hormone) in the stomach is mainly completed by D cells in the stomach and fundus. In the gastric mucosa, it protects and repairs the gastric mucosa by inhibiting the secretion of gastric acid, inhibiting the release of gastrointestinal hormones such as pepsin, motilin, and gastrin, and promoting the healing of gastric injuries. At the same time, SS also participates in the local defense mechanism of gastric mucosa, increases the content of reduced glutathione in cells, improves the ability of gastric mucosa to scavenge oxygen free radicals and lipid peroxides, and reduces gastric mucosal damage (23). These indicators have been used by many researchers to indicate gastric mucosal damage (24). Our results show that deer oil can significantly increase the content of E2, EGF, and SS in the gastric tissue of rats and enhance the defense and repair functions of the gastric mucosa to protect the gastric mucosa.

The pathogenesis of gastric mucosal injury is related to cell apoptosis. The current findings indicate that ethanol triggers gastric apoptosis, which is manifested by the cleavage of caspase-3. The expression of apoptosis-related genes is mainly driven by ROS and proinflammatory signals. In this context, it has been reported that ROS change the protein conformation of pro-apoptotic Bax, causing cytochrome C to escape the mitochondria, thereby activating caspase-3 cleavage (7). Our results show that deer oil can inhibit the cleavage of caspase-3 and the expression of Bax protein, increase the expression of Bcl-2 protein, and reduce cellular apoptosis, thereby playing a role in reducing the gastric damage caused by ethanol.

Acute gastric mucosal injury caused by excessive alcohol leads to the occurrence of inflammation. IL-6, TNF-α, IL-4, and IL-10 are all important inflammation-related factors that are closely related to the occurrence of acute gastric mucosal injury (25). Erythropoietin (EPO) is a hematopoietic factor synthesized and secreted by the kidneys. It is an endogenous cell-protection and inflammation-regulation molecule induced by inflammation. It is one of the endogenous mechanisms of inflammation regulation in the body, and it can inhibit the expression of proinflammatory factors to a certain extent. EPOR is the EPO receptor on the surface of the target cell membrane. Studies have found that EPOR is expressed on the surface of inflammatory cells (26, 27). When the body secretes proinflammatory factors, the expression levels of EPO and EPOR increase to regulate inflammation. The above results provide clear evidence that the model group of rats secreted more proinflammatory factors than the normal group, leading to increased levels of EPO and EPOR. Deer oil pretreatment significantly limited the production of proinflammatory cytokines, increased the secretion of anti-inflammatory cytokines, and effectively inhibited the inflammation of gastric tissue.

Activation of the MAPK cascade and NF-κB transcription pathway occur in many inflammatory and immunomodulatory diseases. Nuclear factor kappa B is a transcription factor that can regulate the expression of a variety of inflammatory mediators, such as TNF-α, IL-6, and IL1β. Pathological disorders of NF-κB signaling are related to the occurrence and development of inflammation, related autoimmune diseases, and cancer (28). Ethanol as an irritant can induce inflammation of gastric mucosal epithelial cells and activate the NF-κB pathway. Studies have shown that the protective effect of Dendrobium nobile polysaccharide on gastric mucosal injury and its mechanism occur through inhibiting the activation of NF-κB and downregulating the ratio of Bax/Bcl2 in gastric mucosa to inhibit cellular apoptosis induced by oxidative stress (29). This is consistent with the results of our study. MAPK, the common pathway of information transmission in cells, participates in the process of extracellular signals from the surface to the inside of the cell. The three main subgroups of MAPK, ERK, JNK, and p38MAPK, require phosphorylation and activation of upstream kinases to perform their biological functions. In the MAPK family, the activation of the main subgroup will regulate the expression of proinflammatory mediators, a phenomenon that has been reported in ethanol-induced gastric ulcers (30). In this study, we found that deer oil pretreatment can prevent the activation of ERK, JNK, p38MAPK, and IKK/IκBα/NF-κB p65 after ethanol stimulation to a certain extent. Therefore, deer oil can inhibit ethanol-induced acute gastric mucosal injury in rats, and its mechanism of action may be through inhibiting the activation of MAPK signaling pathways, thereby inhibiting the nuclear translocation of NF-κB and the expression of downstream inflammatory cytokines. In turn, the occurrence of gastric mucosal damage is inhibited.

In summary, the deer oil extracted by the aqueous enzymatic method is of good quality, has little effect on the active ingredients, and has a certain protective effect on the acute gastric mucosal injury caused by ethanol.

## Data Availability Statement

The original contributions presented in the study are included in the article/Supplementary Materials, further inquiries can be directed to the corresponding author/s.

## Ethics Statement

The animal study was reviewed and approved by Institute of Special Animal and Plant, Chinese Academy of Agricultural Sciences.

## Author Contributions

Y-SX performed the animal experiment and manuscript. D-DR and RM performed the date analysis. P-PB, Y-TZ, and L-jZ collected the samples. L-jZ and CL supervised the research. Y-sS and ZW designed the experiment. All authors listed have made a substantial, direct and intellectual contribution to the work, and approved it for publication.

## Conflict of Interest

The authors declare that the research was conducted in the absence of any commercial or financial relationships that could be construed as a potential conflict of interest.

## Publisher's Note

All claims expressed in this article are solely those of the authors and do not necessarily represent those of their affiliated organizations, or those of the publisher, the editors and the reviewers. Any product that may be evaluated in this article, or claim that may be made by its manufacturer, is not guaranteed or endorsed by the publisher.
