# Supplementary figures and images for: Effect of Aqueous Enzymatic Extraction of Deer Oil on Its Components and Its Protective Effect on Gastric Mucosa Injury
Source: Front Nutr. 2021 Nov 16;8:769463. doi: 10.3389/fnut.2021.769463 (PMC8635026; doi:10.3389/fnut.2021.769463)

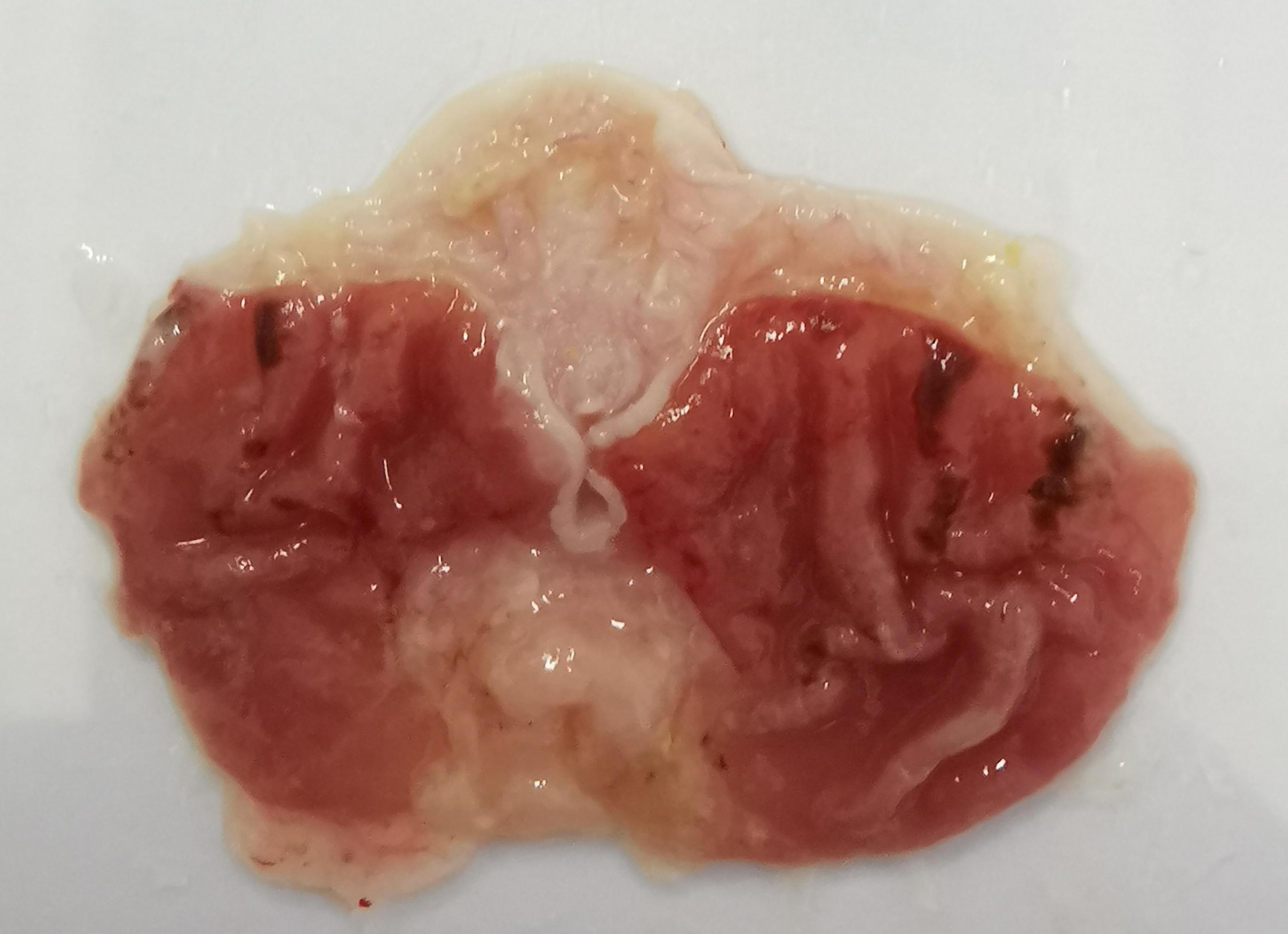

Supplement: Supplementary file 2 [file Data_Sheet_1.ZIP › HE/Cimetidine.1jpg.jpg]

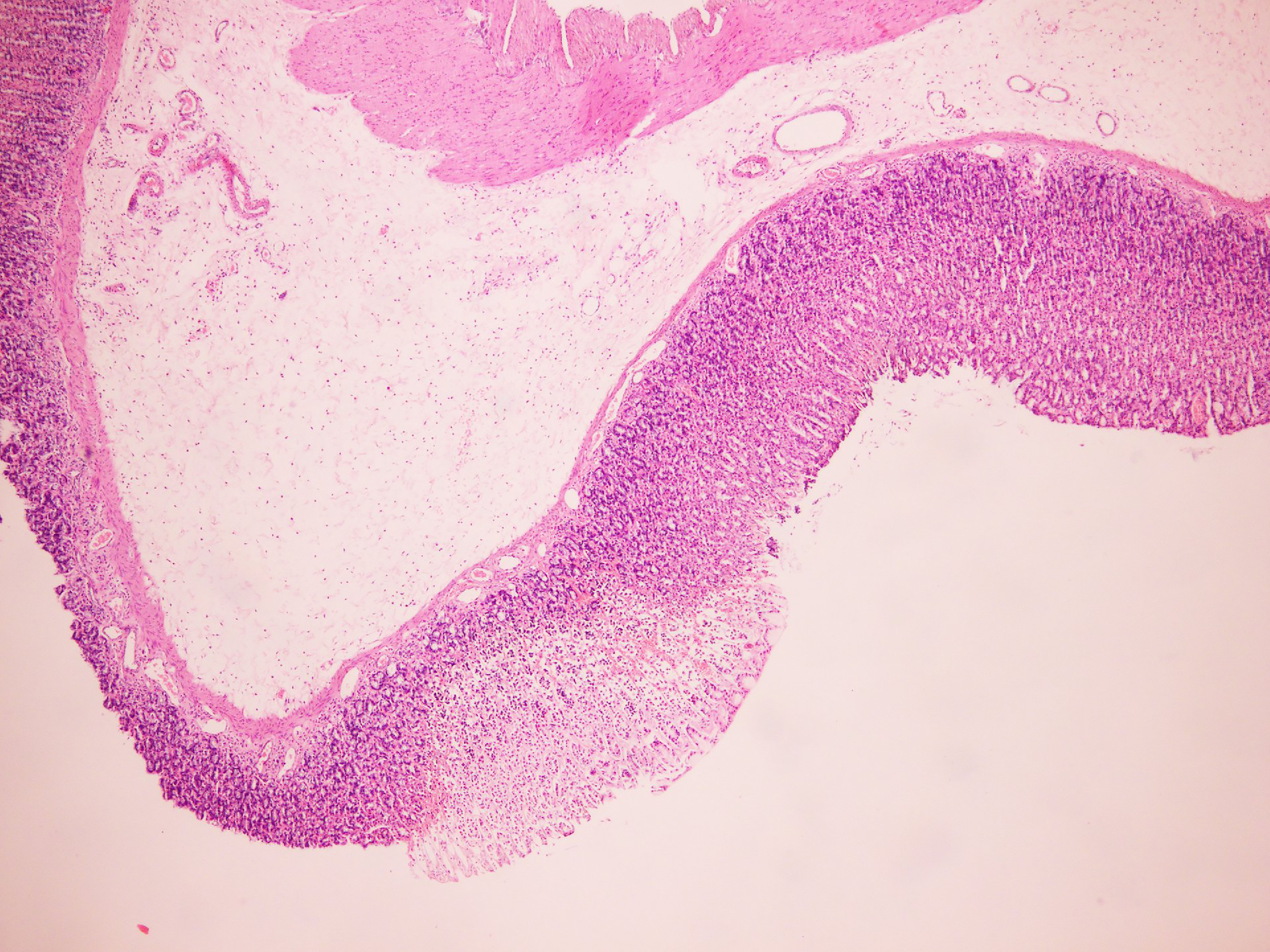

Supplement: Supplementary file 2 [file Data_Sheet_1.ZIP › HE/Cimetidine.jpg]

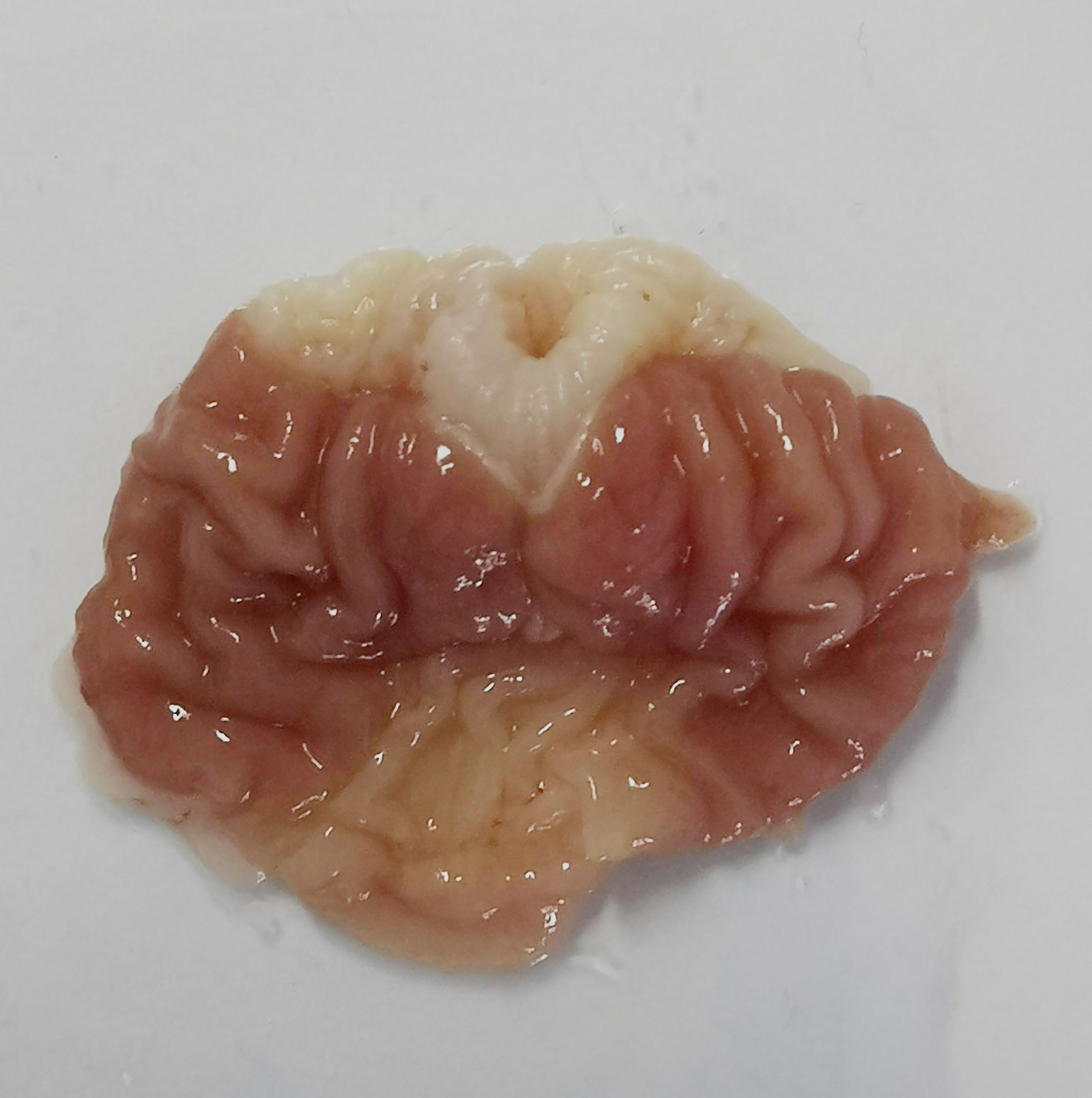

Supplement: Supplementary file 2 [file Data_Sheet_1.ZIP › HE/control.1jpg.jpg]

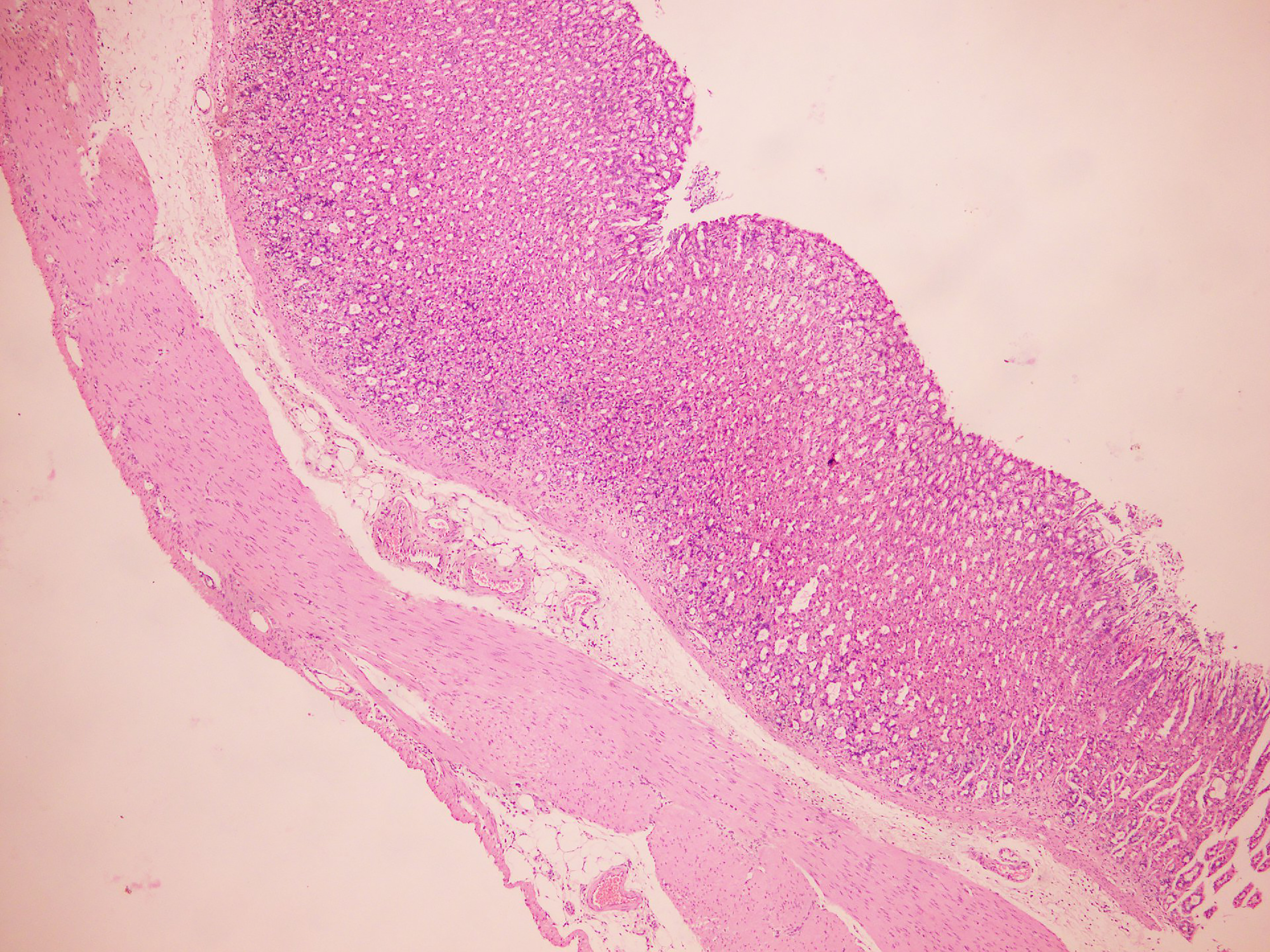

Supplement: Supplementary file 2 [file Data_Sheet_1.ZIP › HE/Control.jpg]

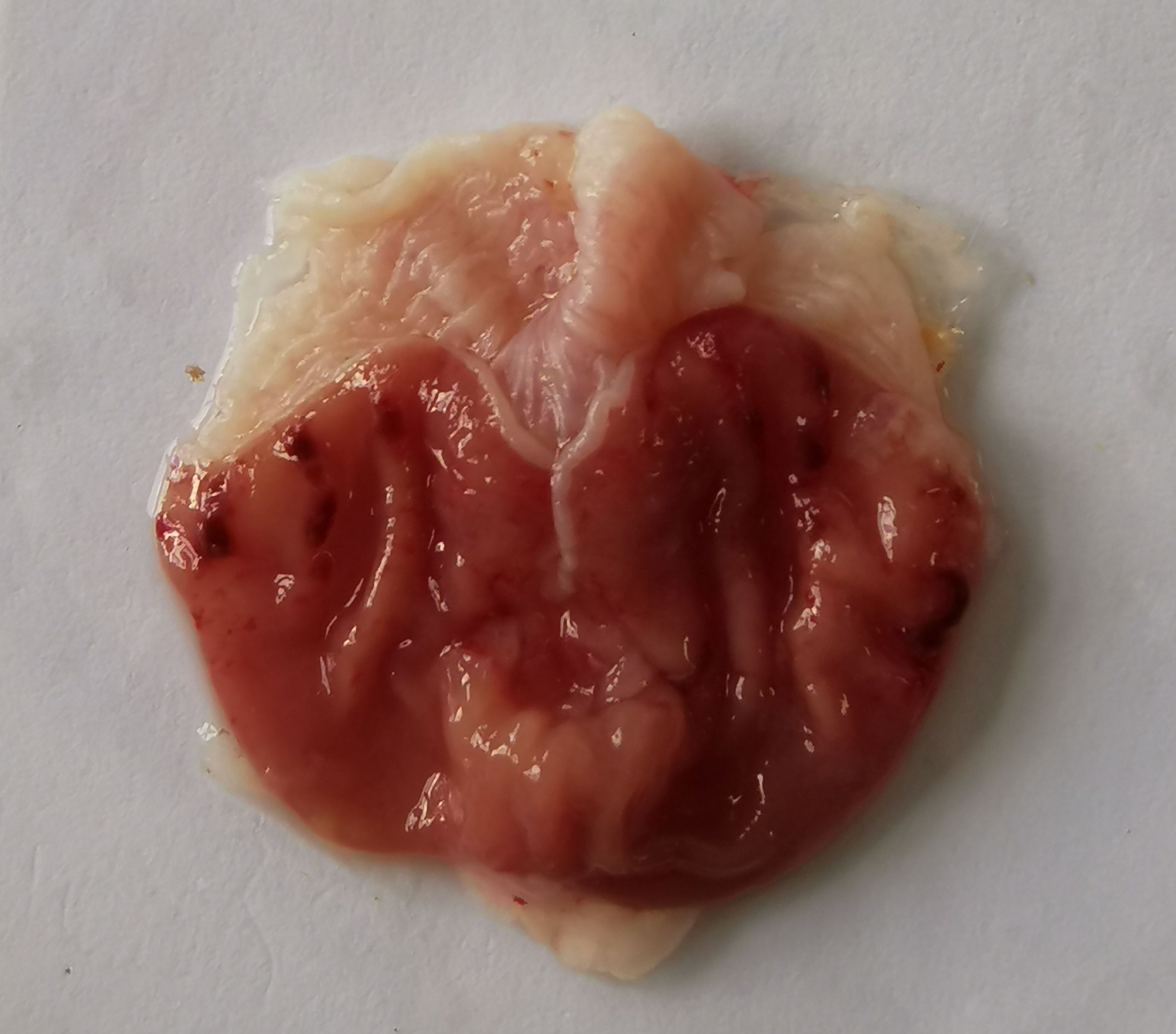

Supplement: Supplementary file 2 [file Data_Sheet_1.ZIP › HE/H-deer oil.1jpg.jpg]

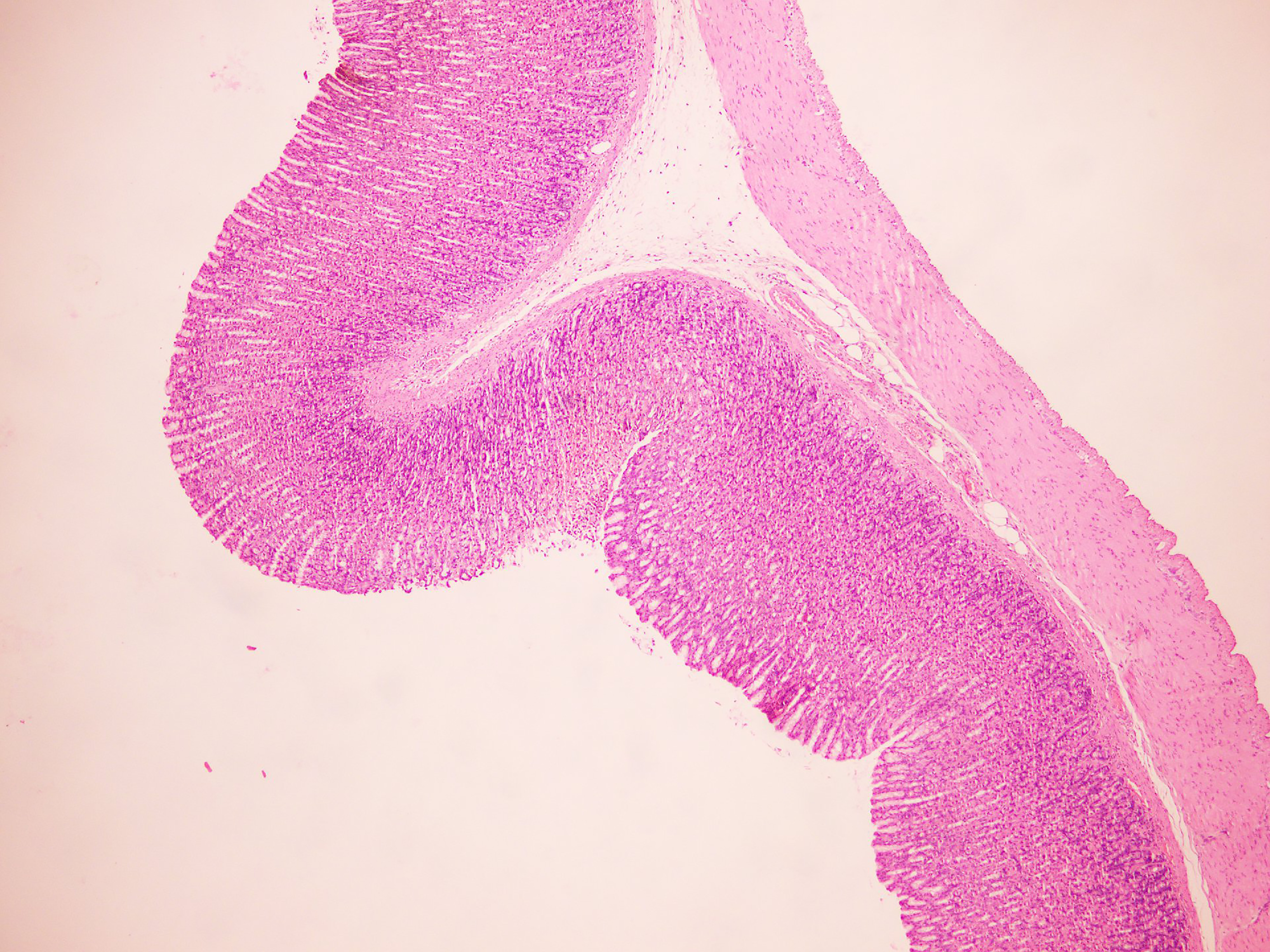

Supplement: Supplementary file 2 [file Data_Sheet_1.ZIP › HE/Hdeer oil.jpg]

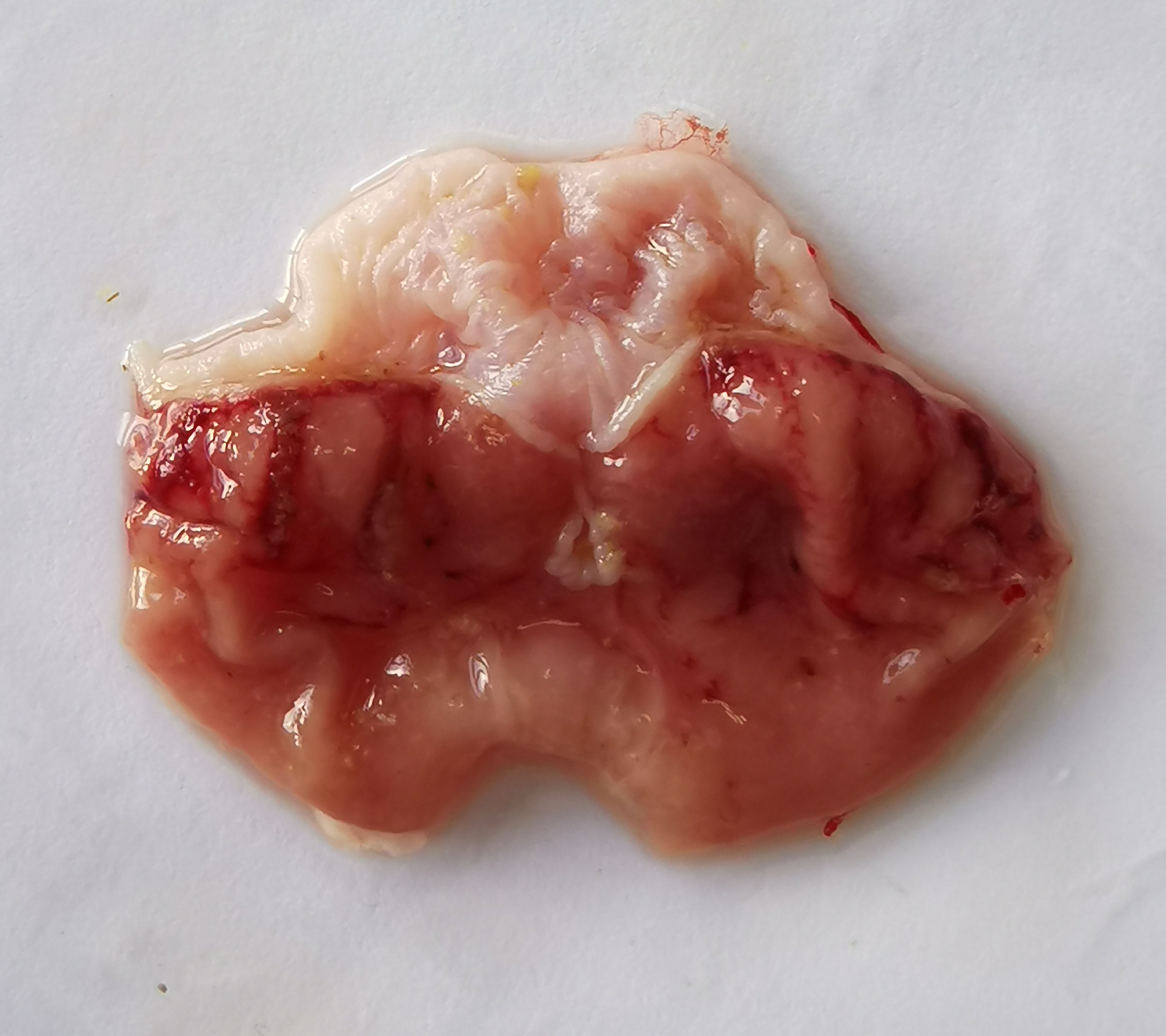

Supplement: Supplementary file 2 [file Data_Sheet_1.ZIP › HE/l-deer oil.1jpg.jpg]

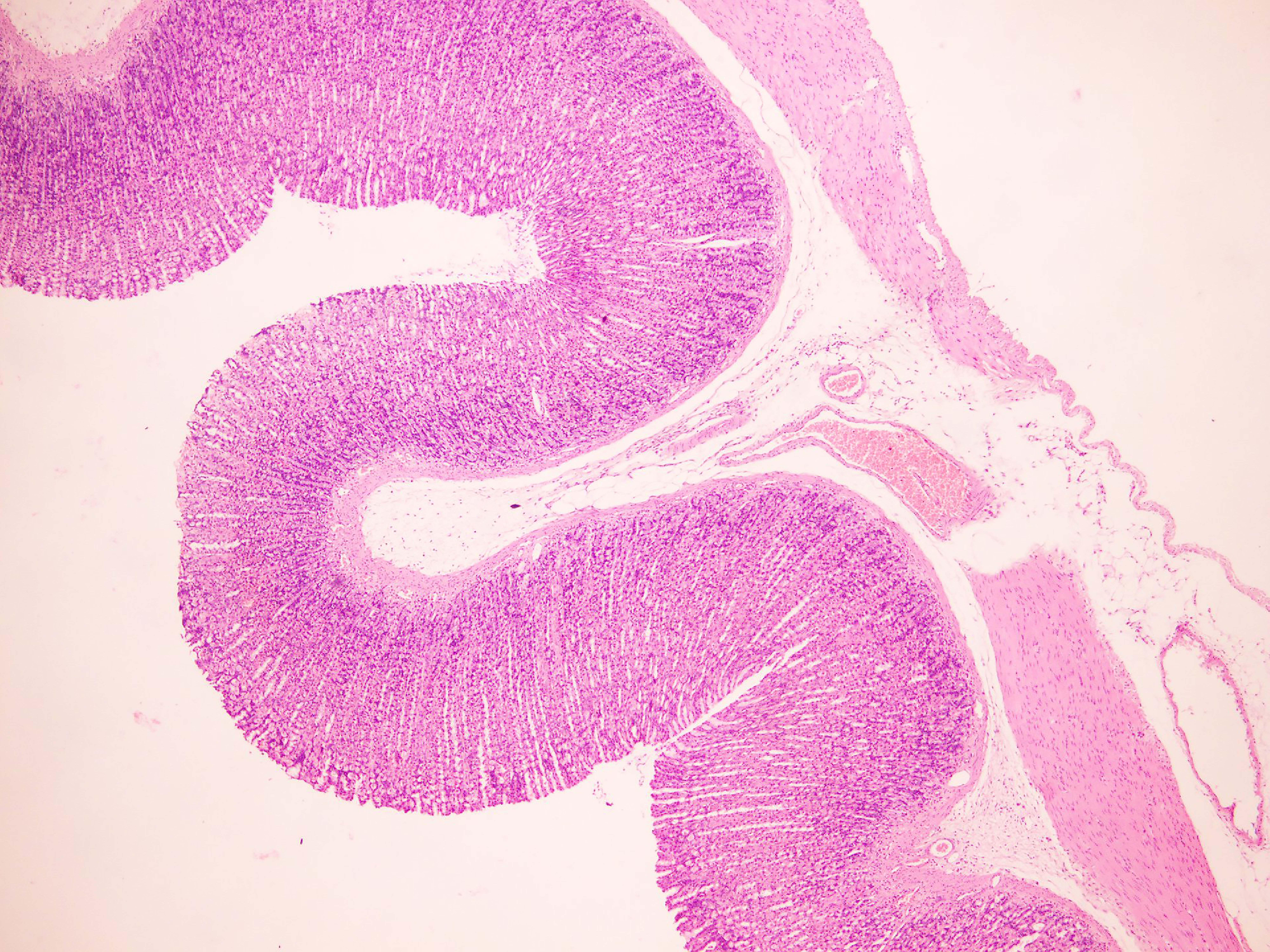

Supplement: Supplementary file 2 [file Data_Sheet_1.ZIP › HE/L-deer oil.jpg]

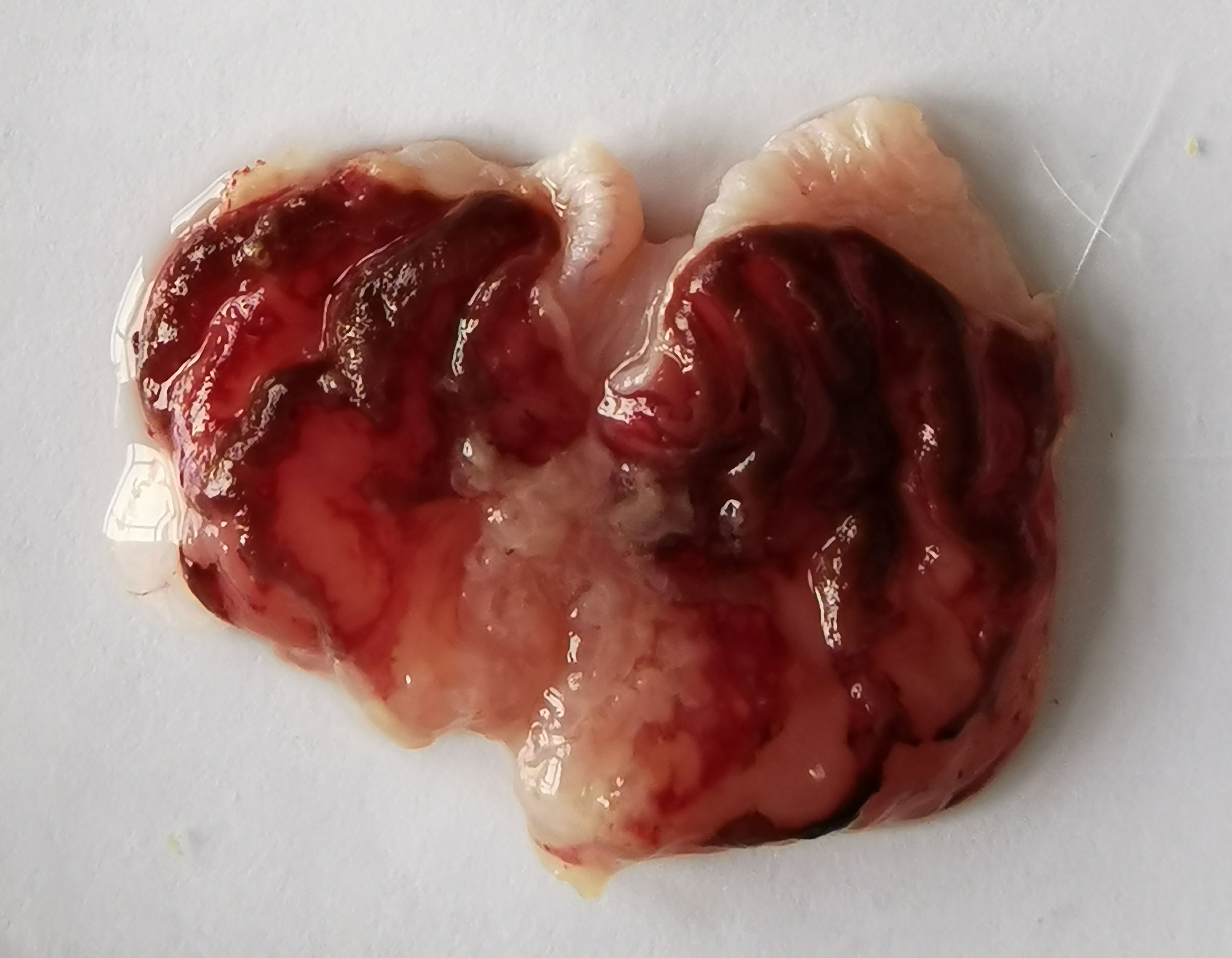

Supplement: Supplementary file 2 [file Data_Sheet_1.ZIP › HE/Model.1jpg.jpg]

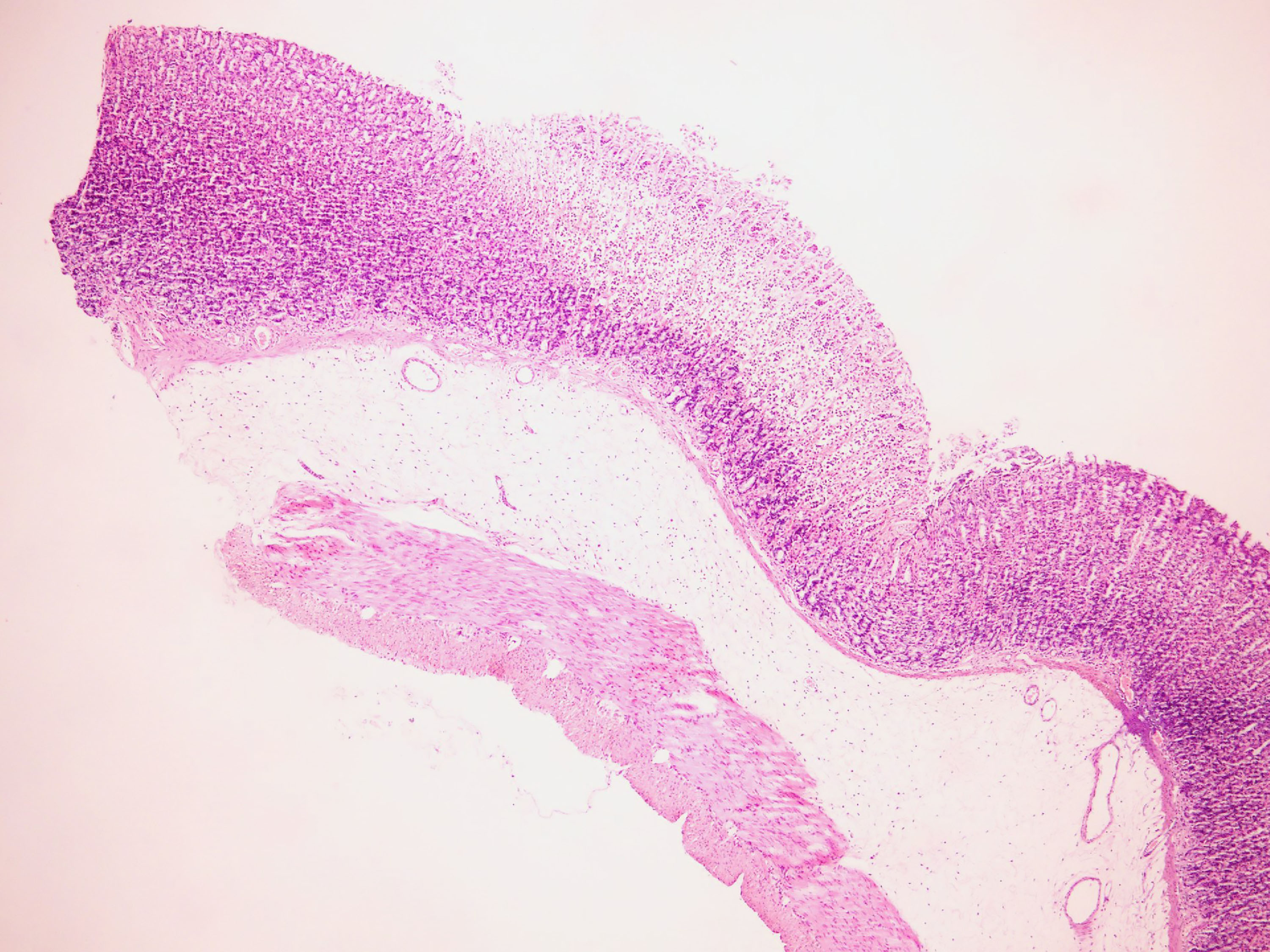

Supplement: Supplementary file 2 [file Data_Sheet_1.ZIP › HE/Model.jpg]

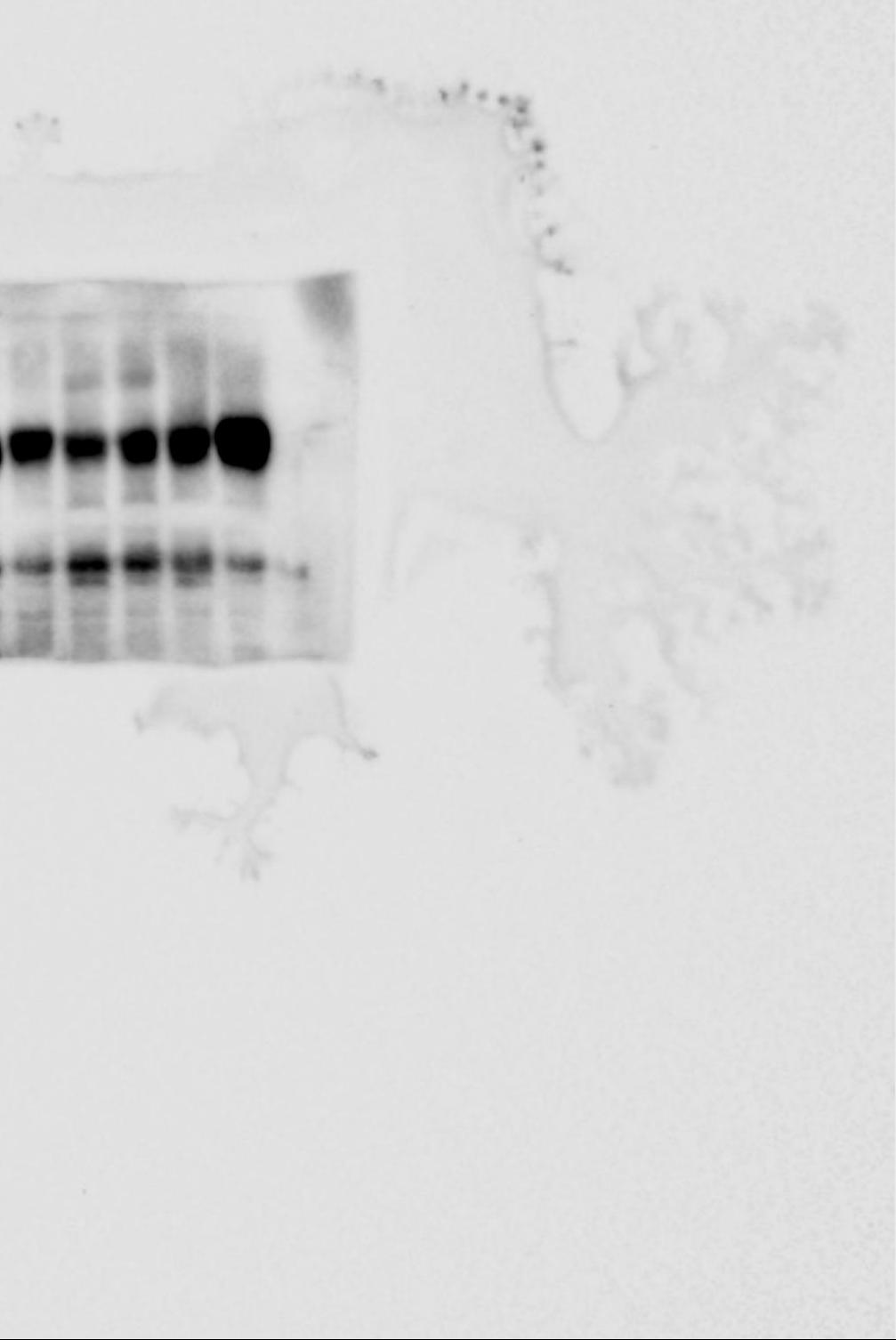

Supplement: Supplementary file 3 [file Data_Sheet_2.ZIP › The original image/bax (2).jpg]

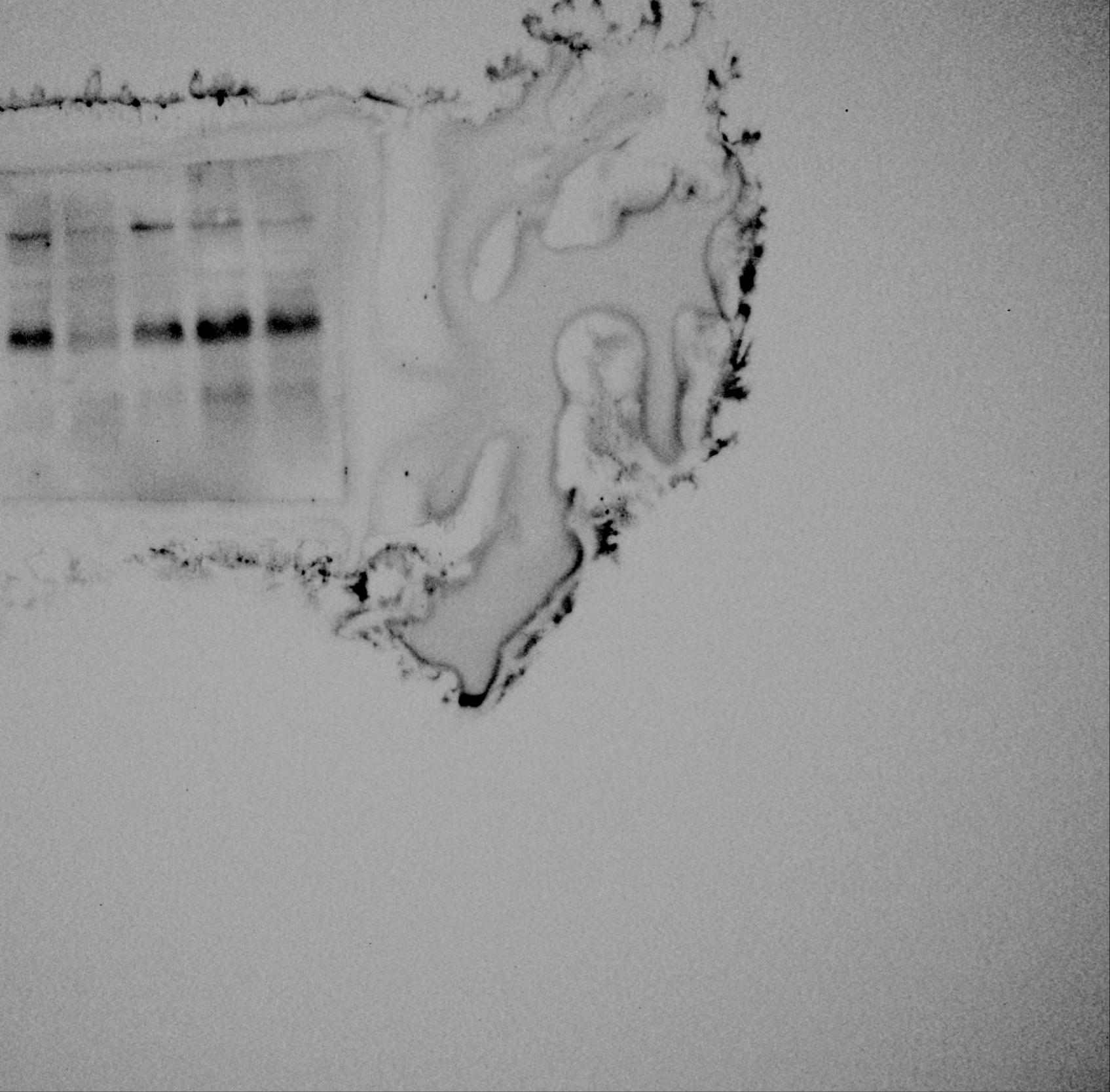

Supplement: Supplementary file 3 [file Data_Sheet_2.ZIP › The original image/bcl-2(2).jpg]

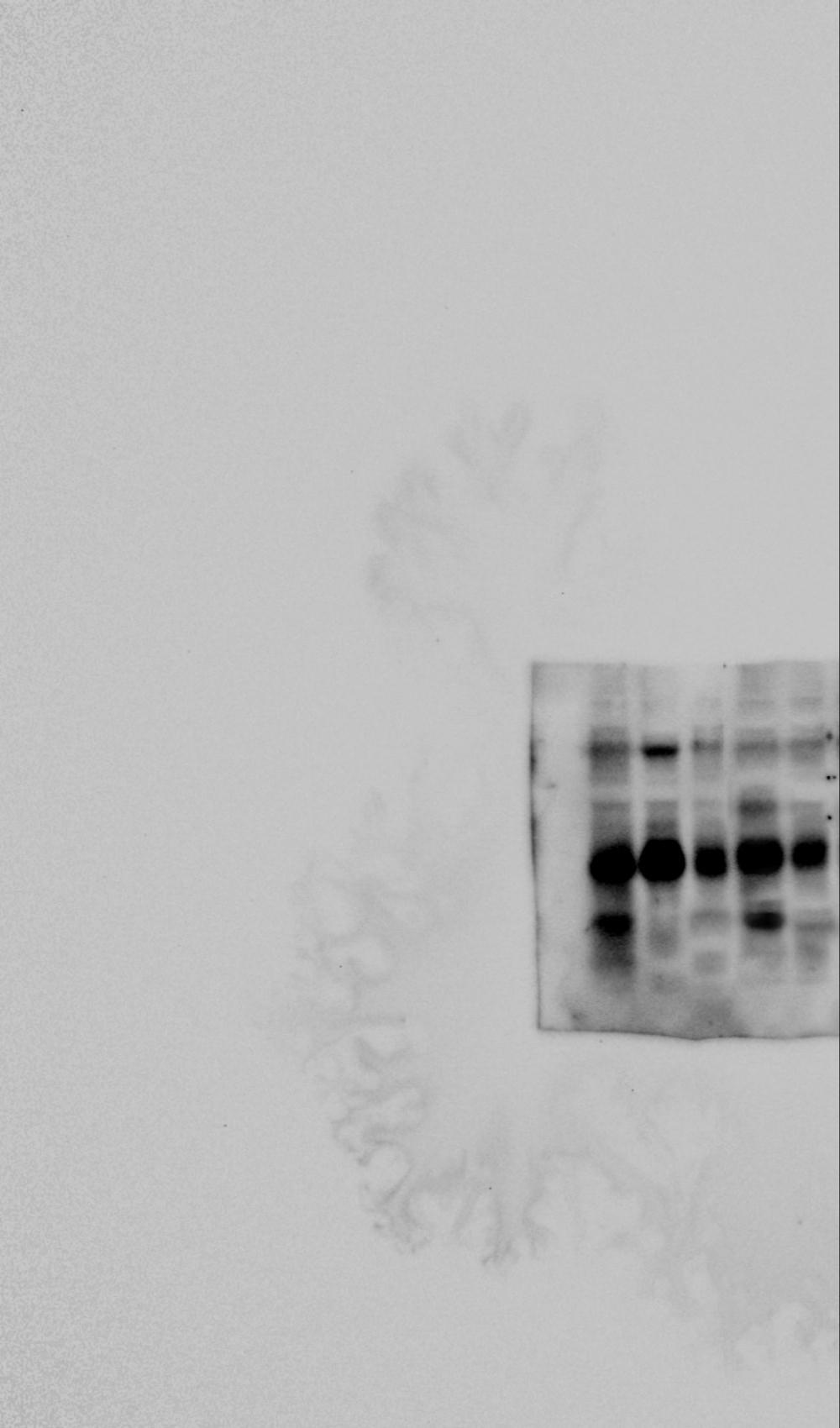

Supplement: Supplementary file 3 [file Data_Sheet_2.ZIP › The original image/cleaved caspase-3(2).jpg]

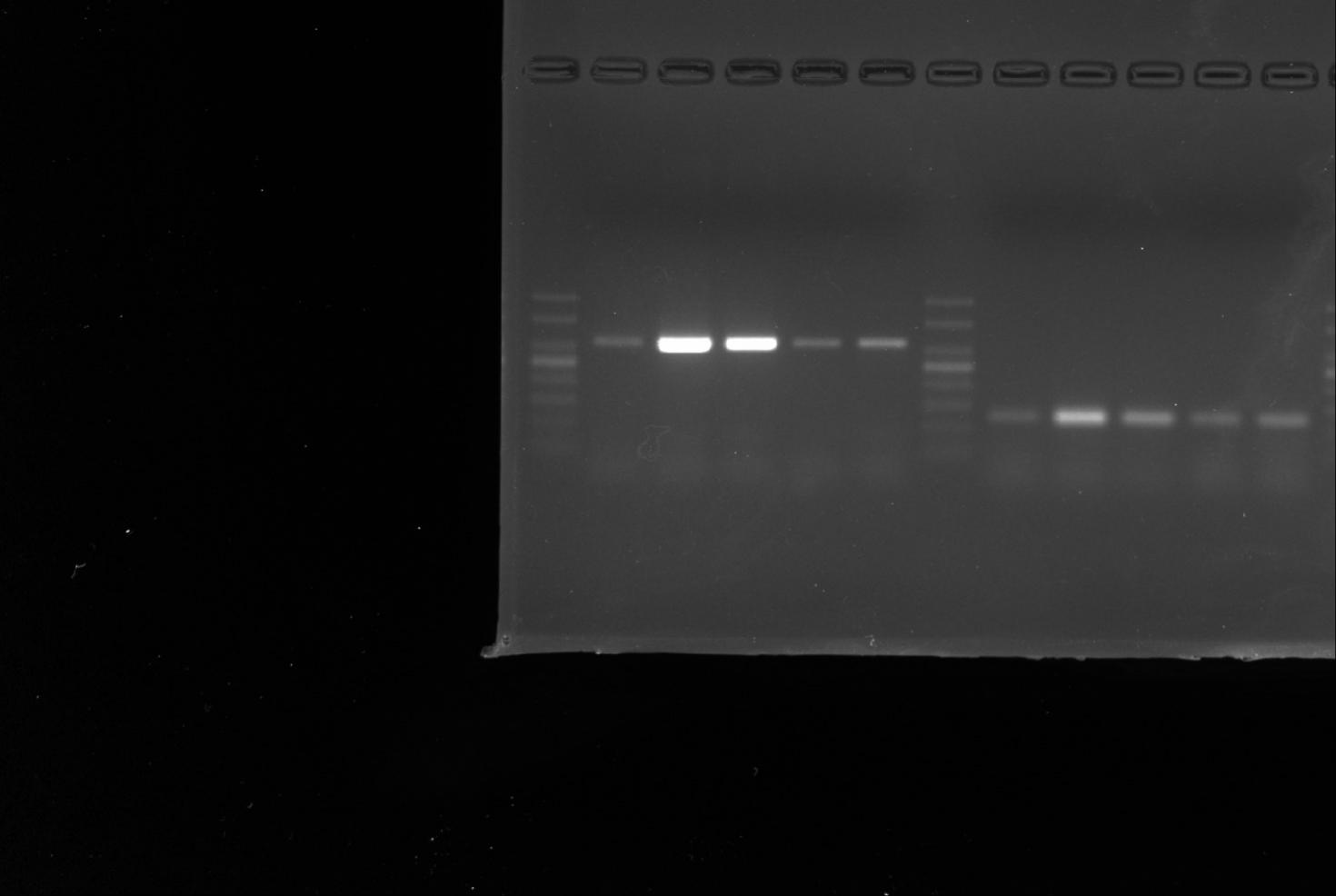

Supplement: Supplementary file 3 [file Data_Sheet_2.ZIP › The original image/EPO;EPOR.jpg]

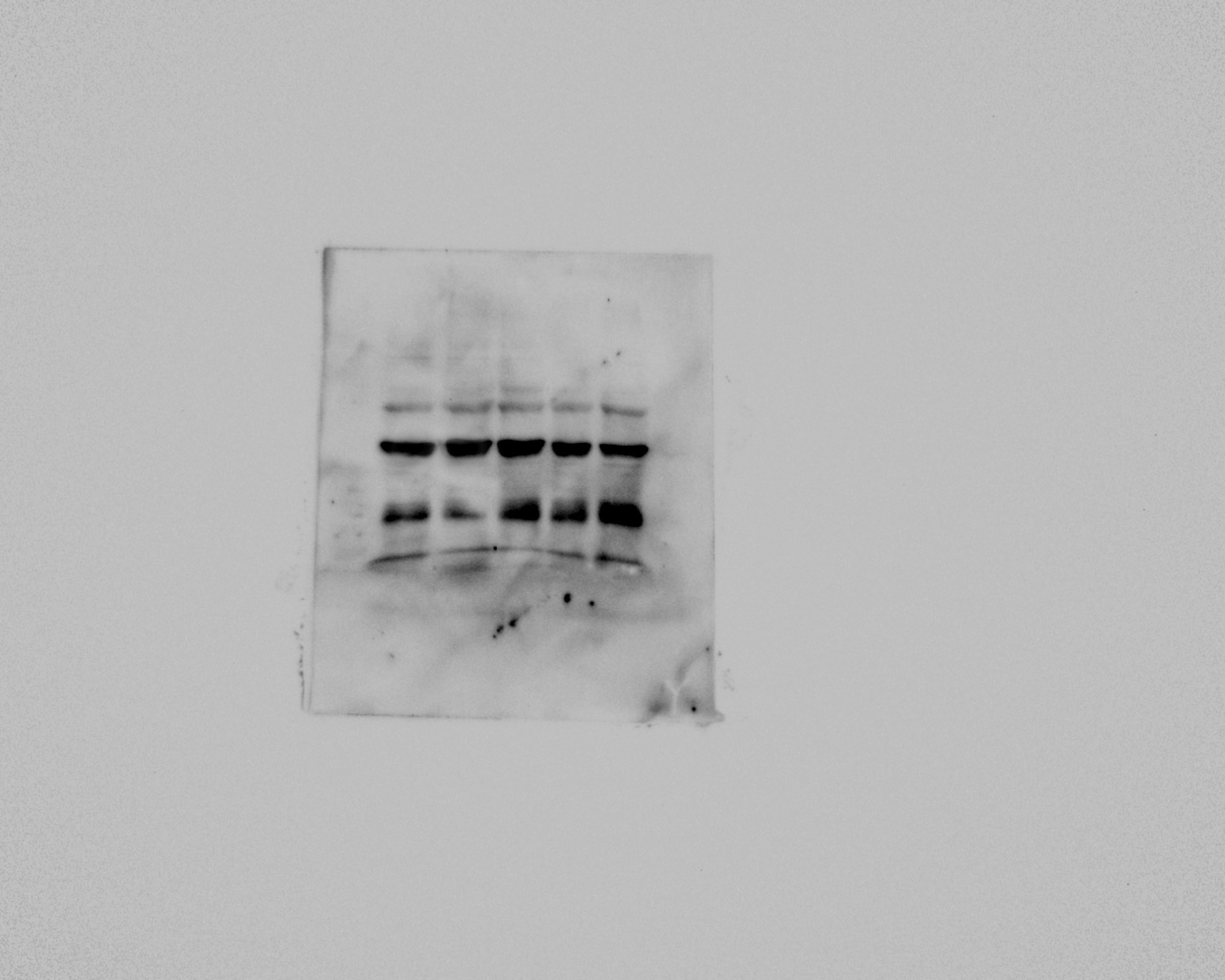

Supplement: Supplementary file 3 [file Data_Sheet_2.ZIP › The original image/erk.jpg]

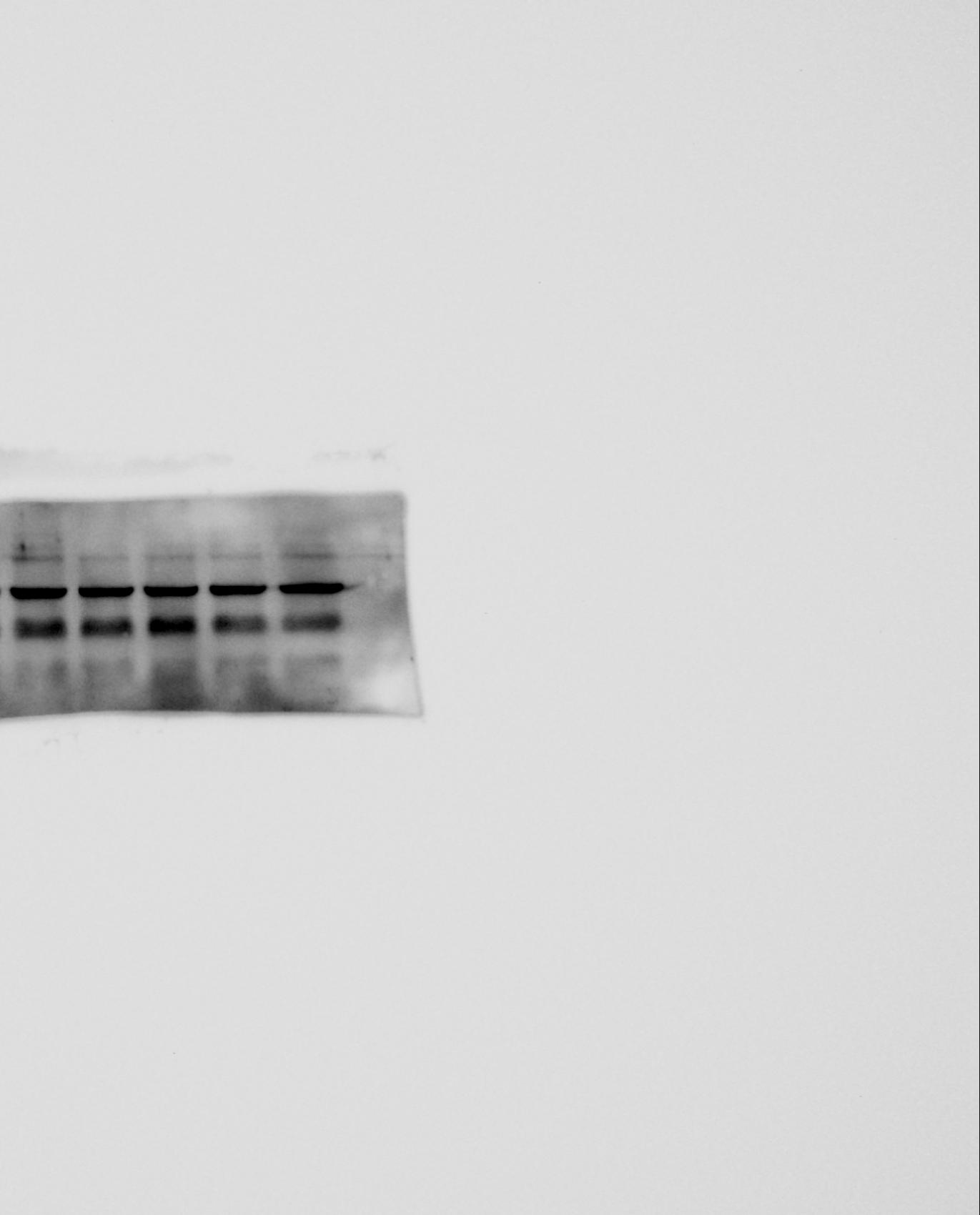

Supplement: Supplementary file 3 [file Data_Sheet_2.ZIP › The original image/GAPDH1.jpg]

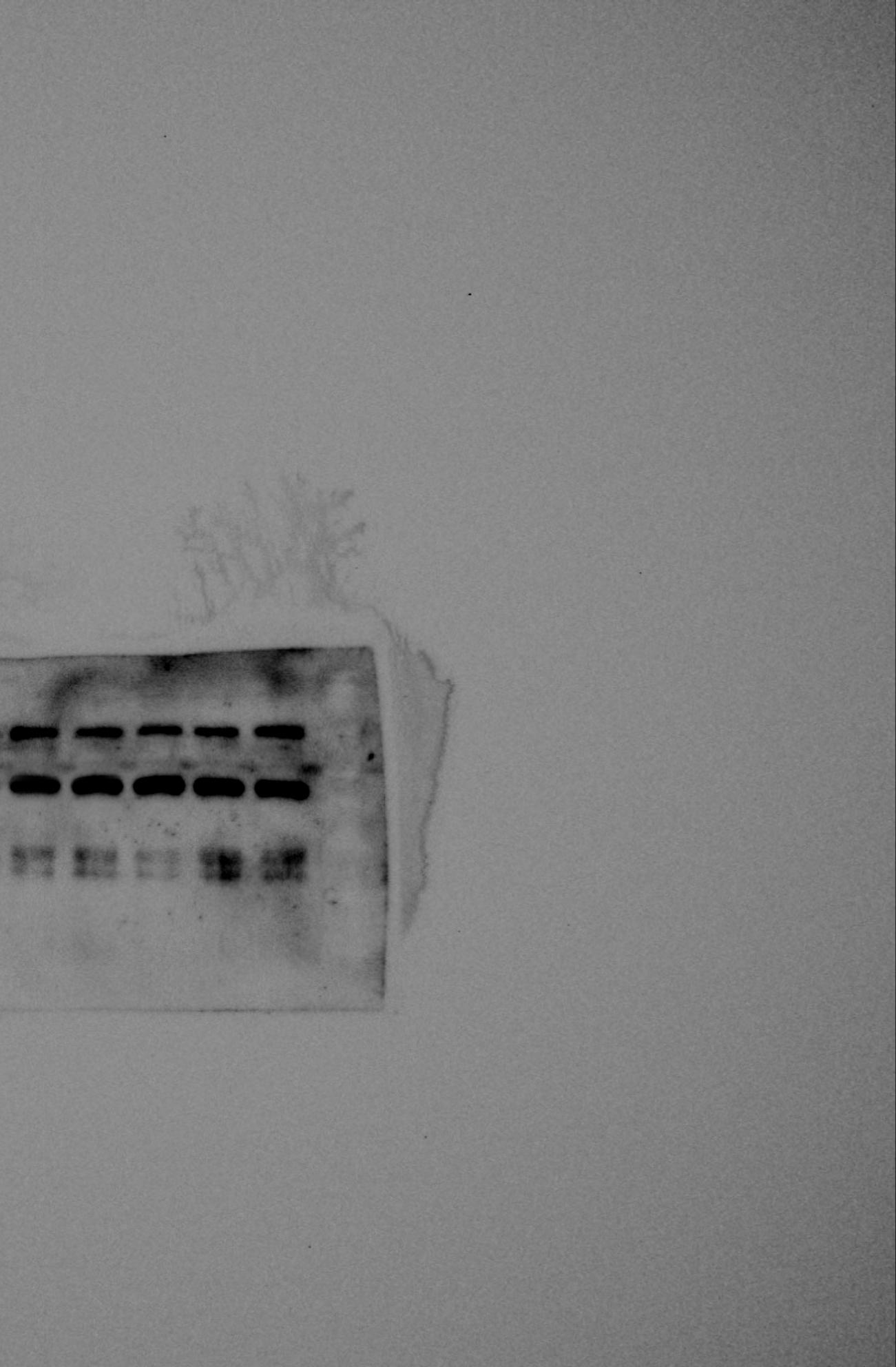

Supplement: Supplementary file 3 [file Data_Sheet_2.ZIP › The original image/GAPDH2.jpg]

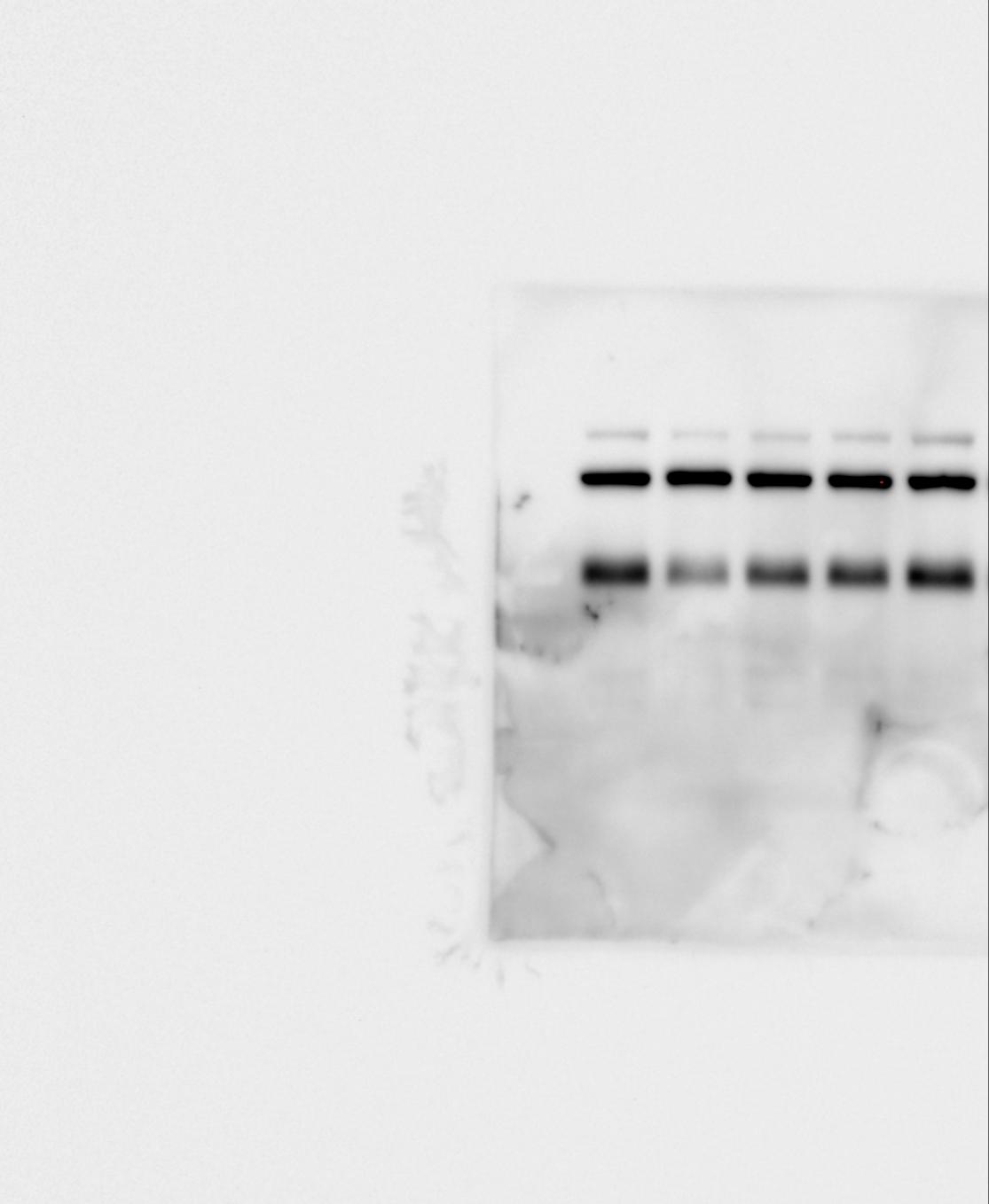

Supplement: Supplementary file 3 [file Data_Sheet_2.ZIP › The original image/GAPDH3.jpg]

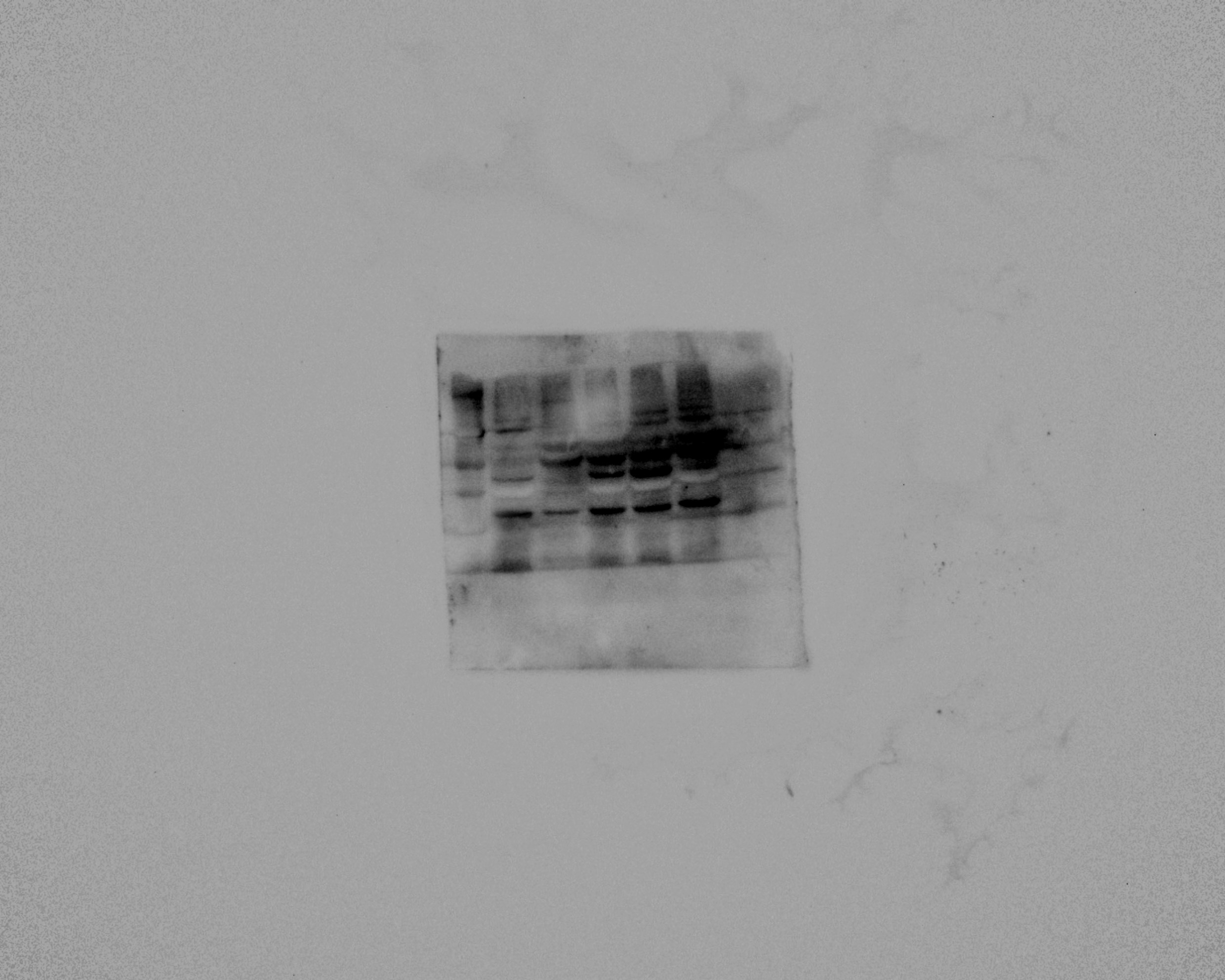

Supplement: Supplementary file 3 [file Data_Sheet_2.ZIP › The original image/ikb-α.jpg]

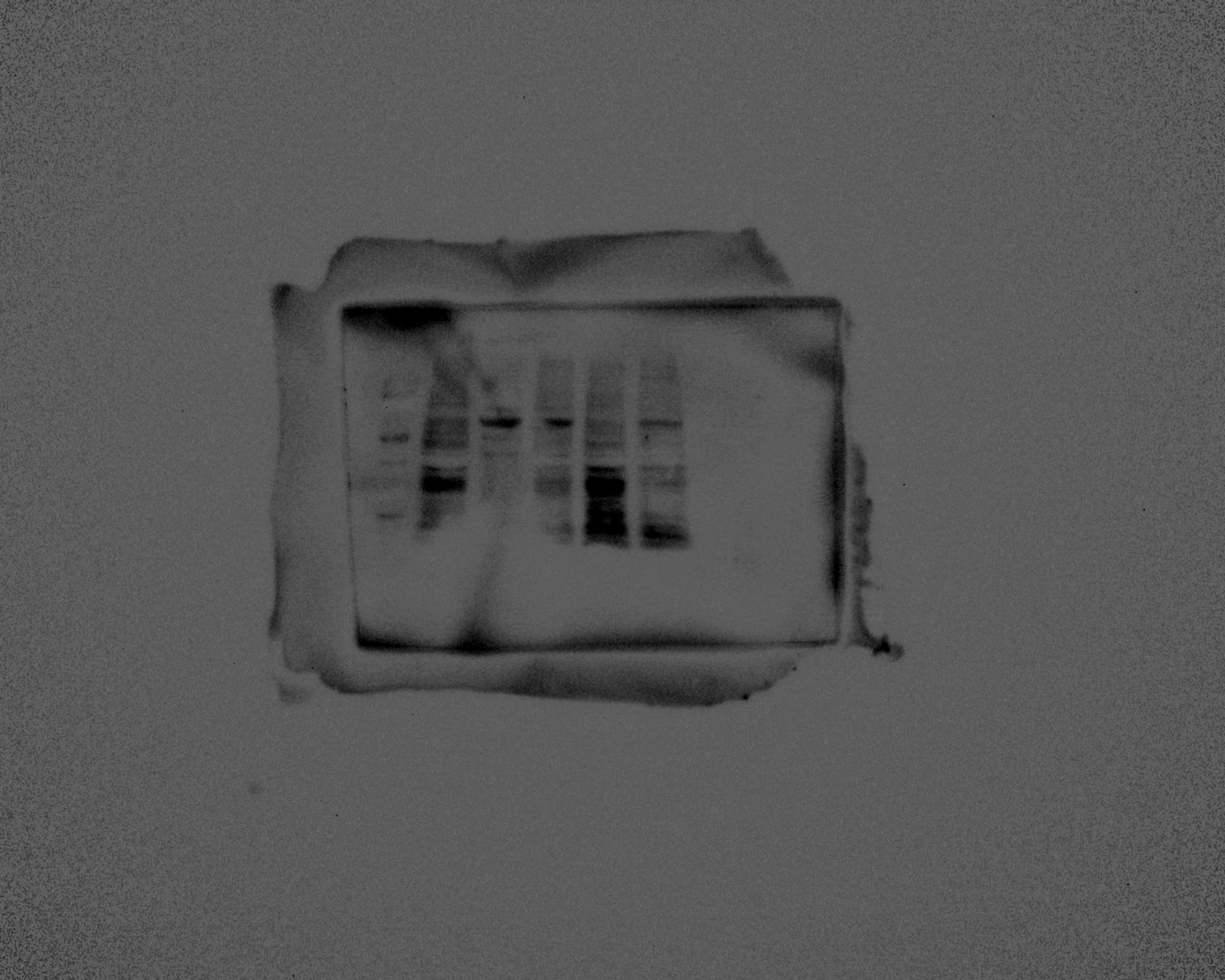

Supplement: Supplementary file 3 [file Data_Sheet_2.ZIP › The original image/ikk.jpg]

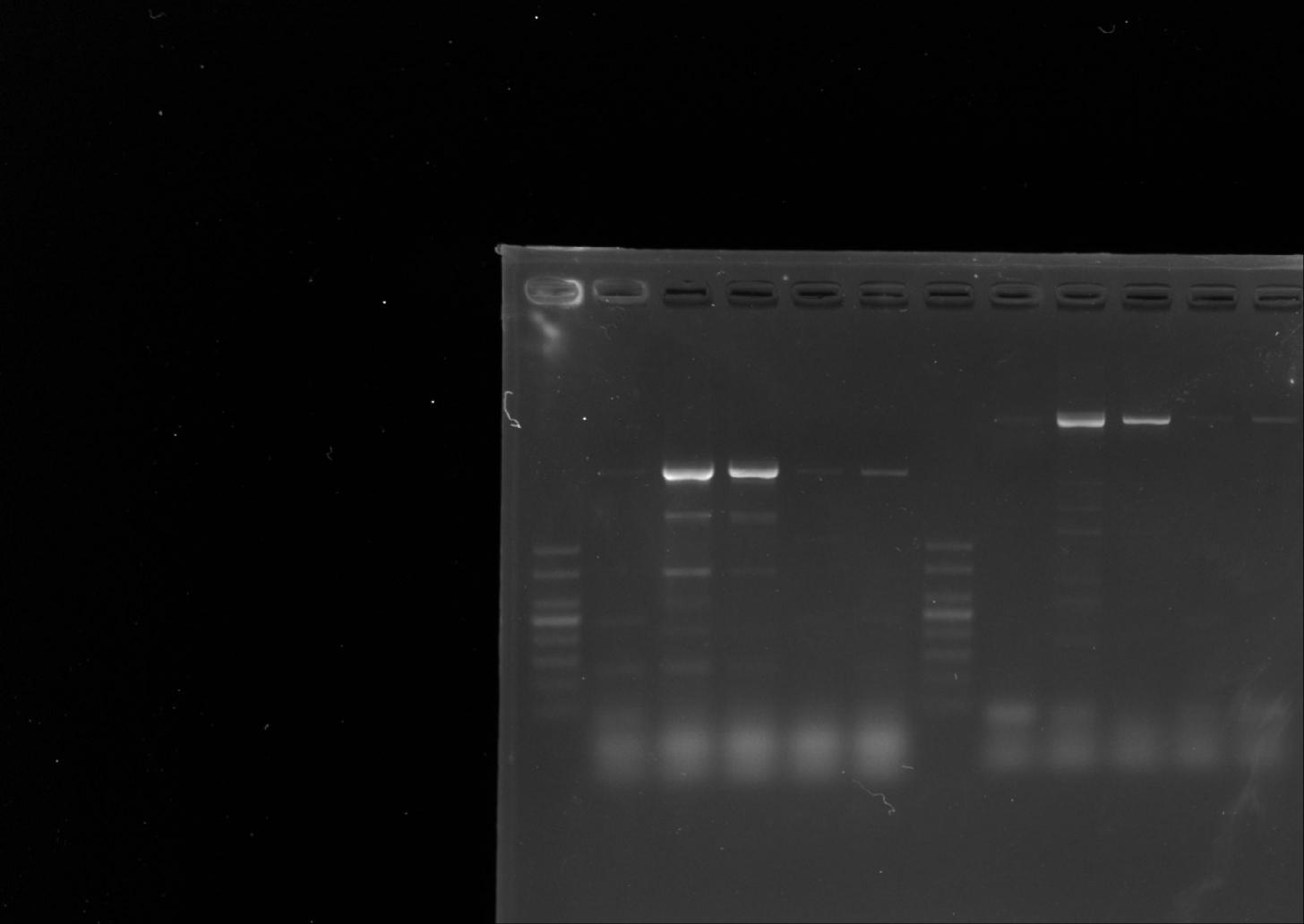

Supplement: Supplementary file 3 [file Data_Sheet_2.ZIP › The original image/IL-1β;IL-6.jpg]

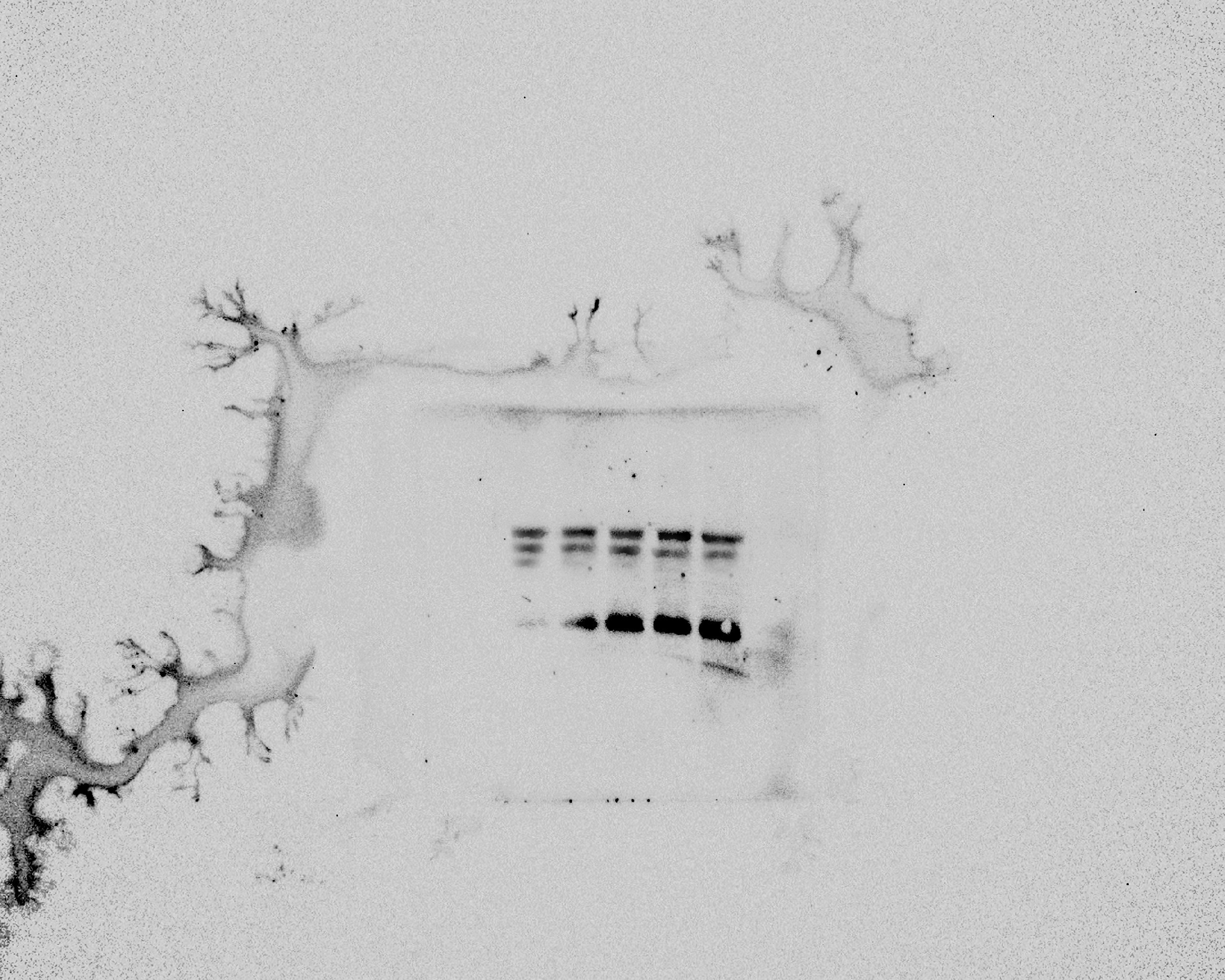

Supplement: Supplementary file 3 [file Data_Sheet_2.ZIP › The original image/JNK.jpg]

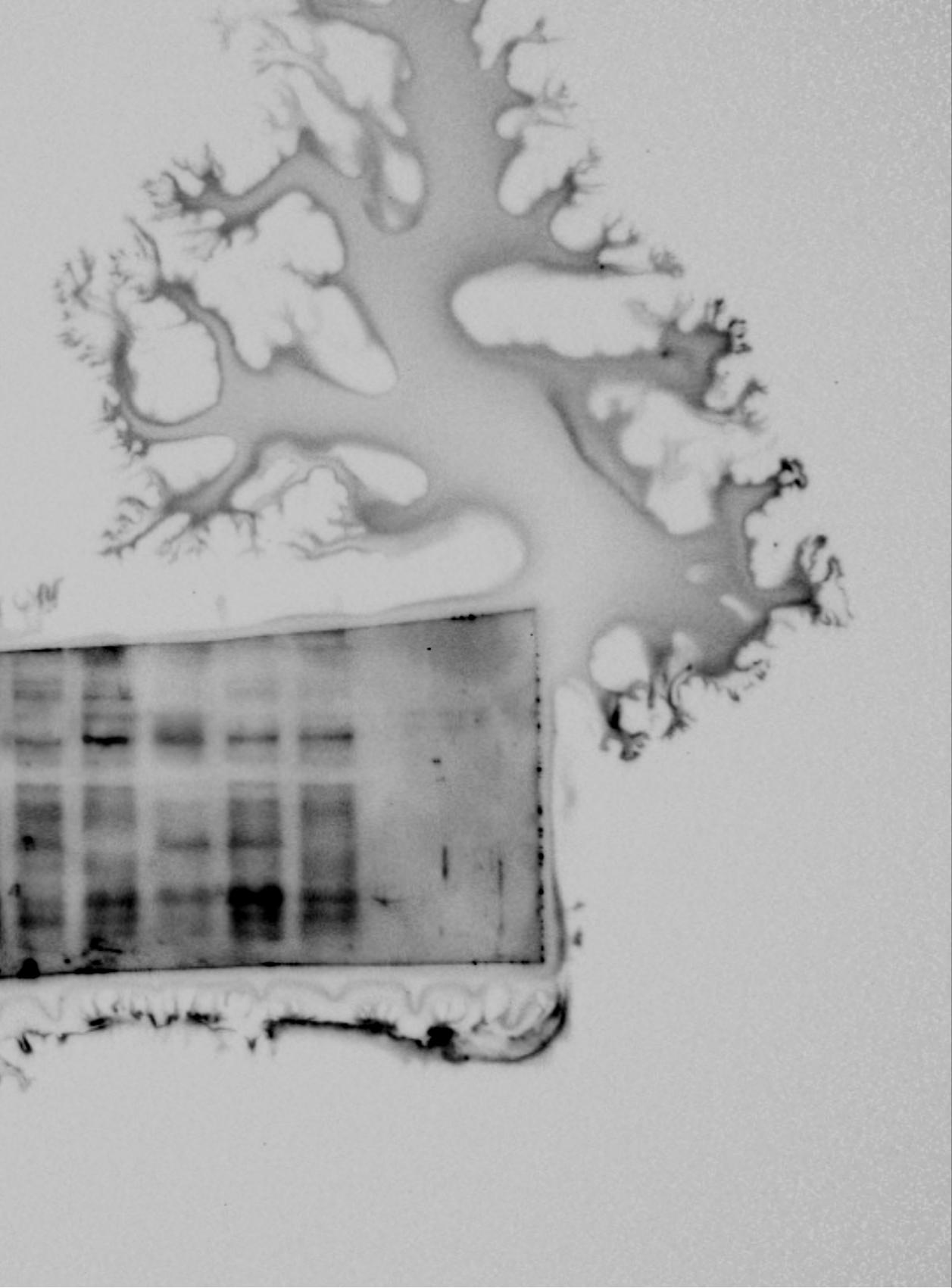

Supplement: Supplementary file 3 [file Data_Sheet_2.ZIP › The original image/nfkb.jpg]

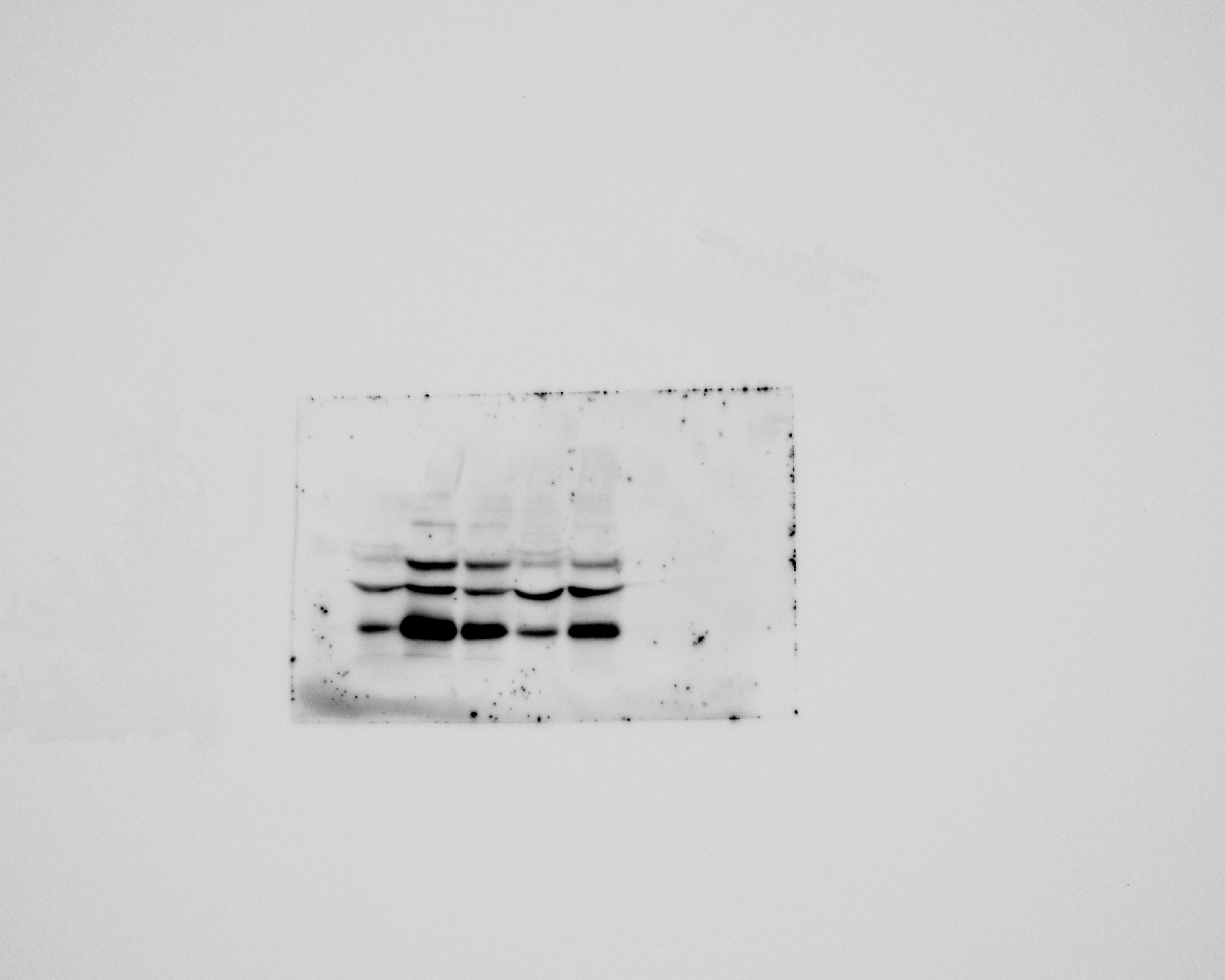

Supplement: Supplementary file 3 [file Data_Sheet_2.ZIP › The original image/p-erk.jpg]

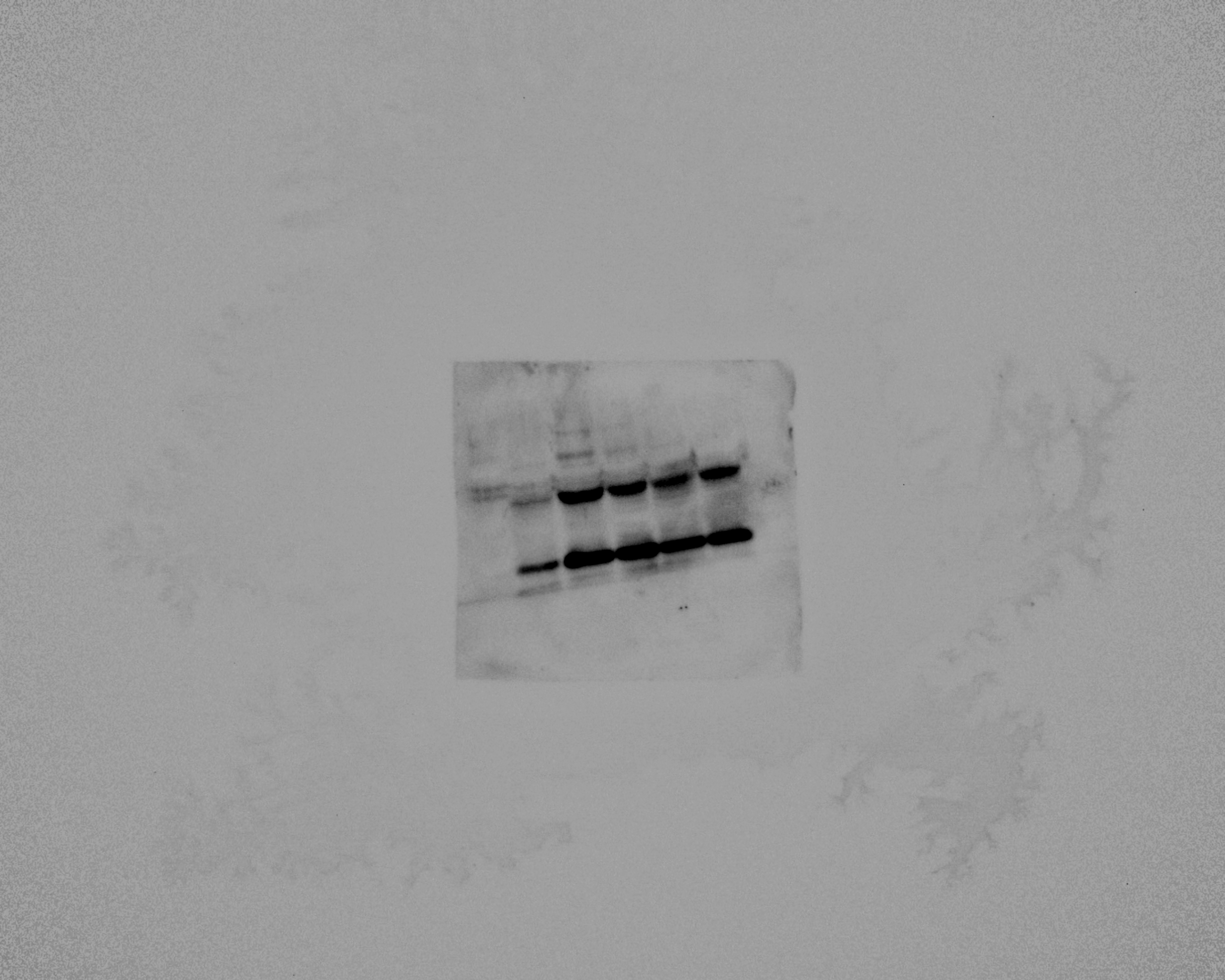

Supplement: Supplementary file 3 [file Data_Sheet_2.ZIP › The original image/p-ikb.jpg]

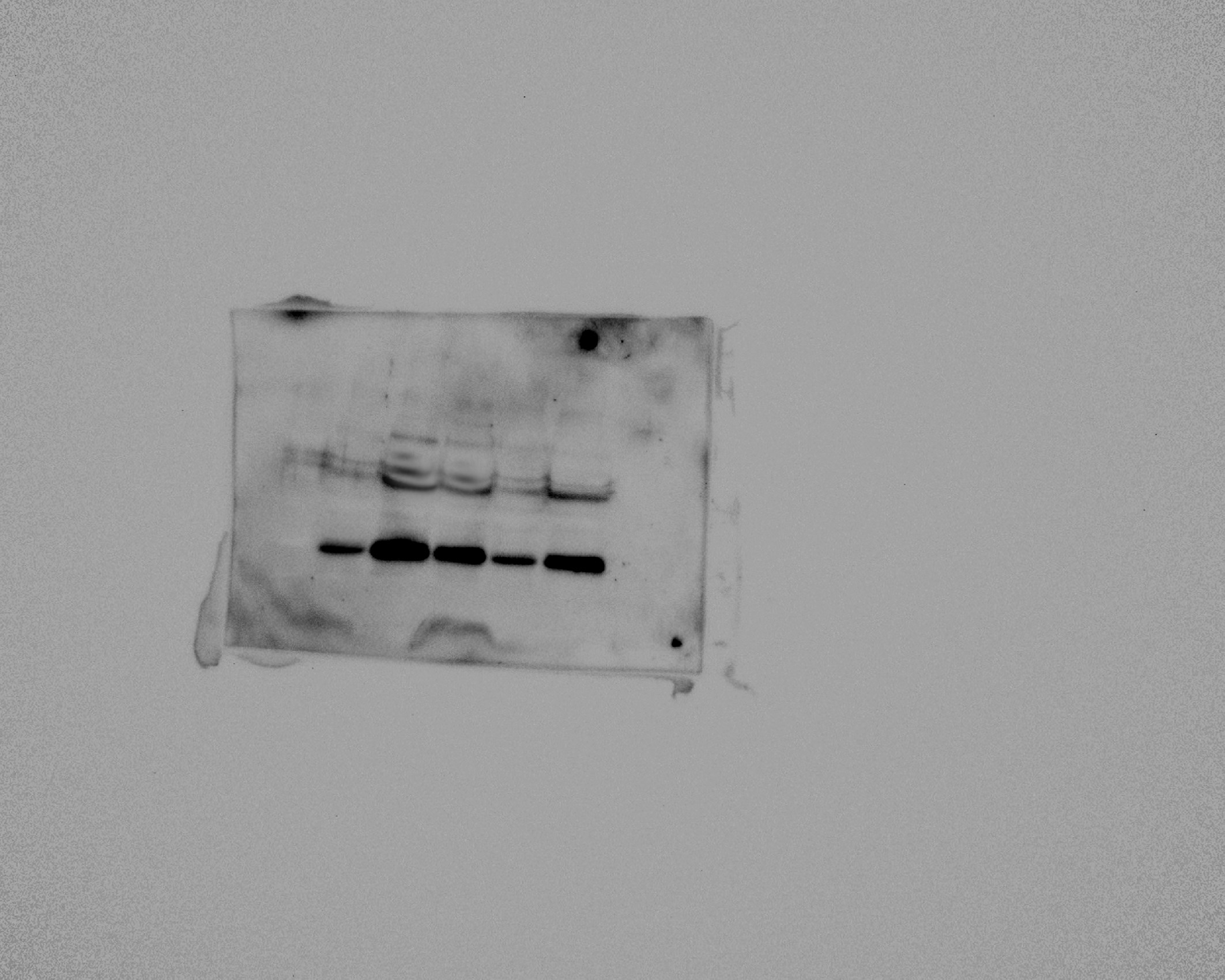

Supplement: Supplementary file 3 [file Data_Sheet_2.ZIP › The original image/p-jnk.jpg]

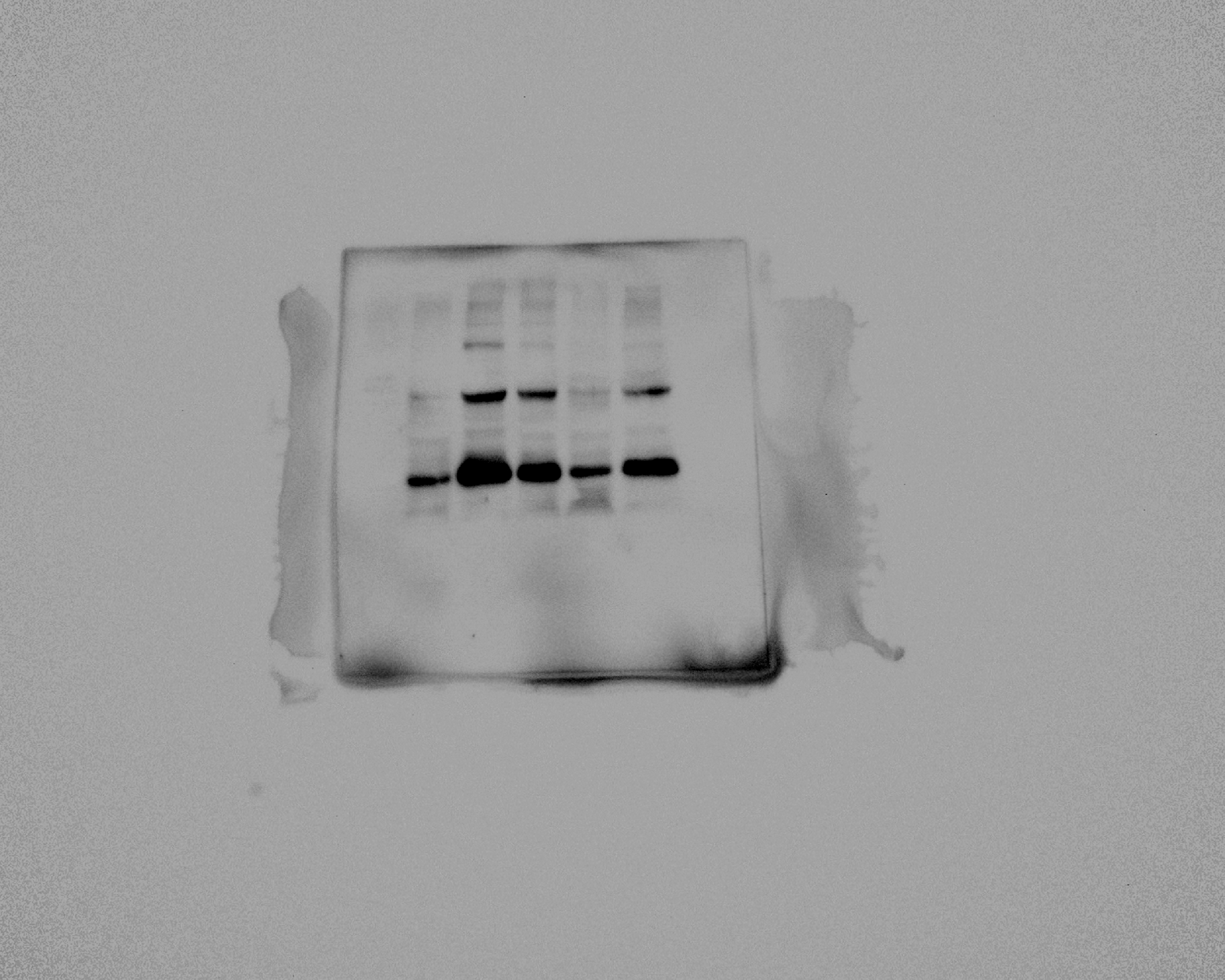

Supplement: Supplementary file 3 [file Data_Sheet_2.ZIP › The original image/p-nfkb.jpg]

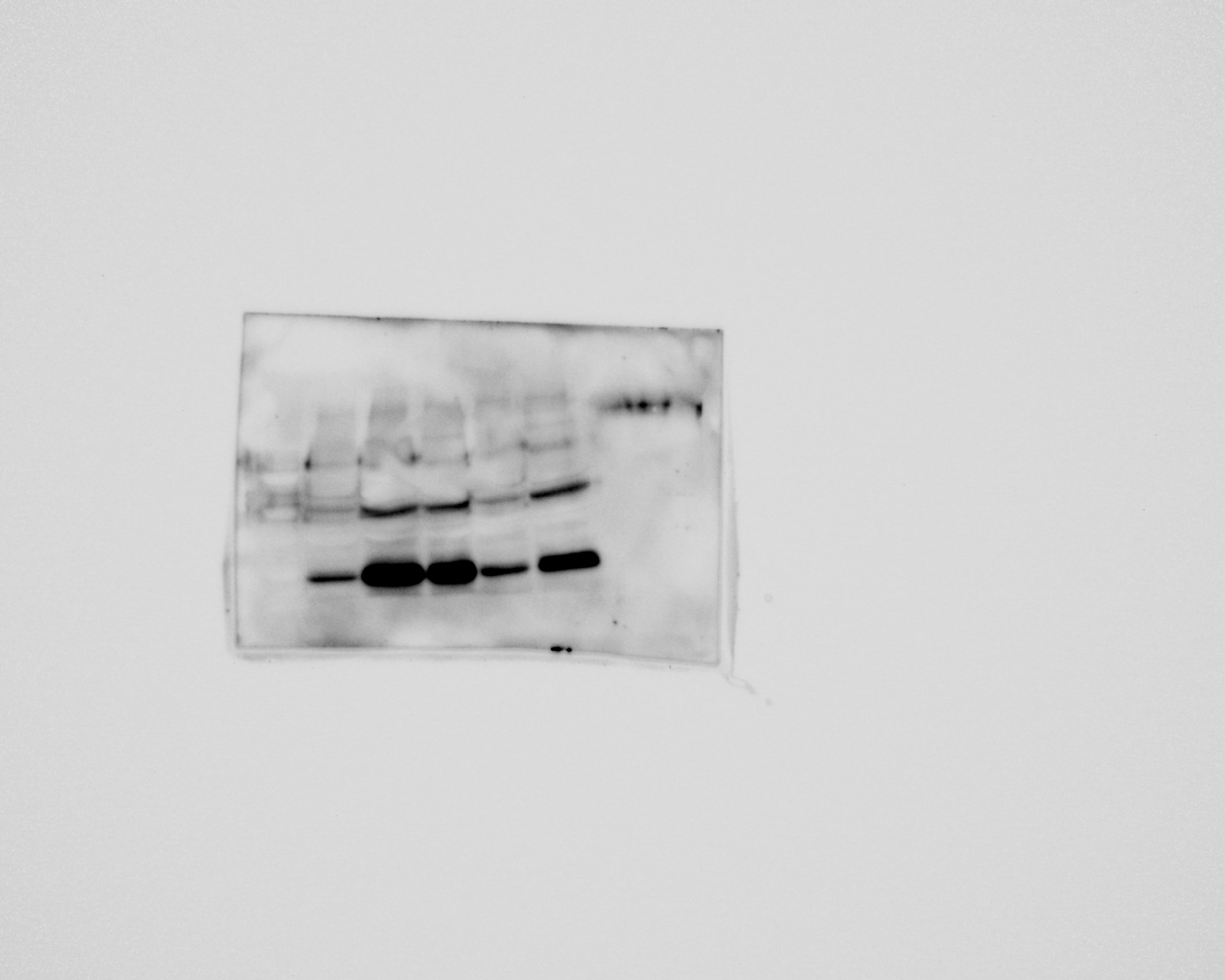

Supplement: Supplementary file 3 [file Data_Sheet_2.ZIP › The original image/p-p38.jpg]

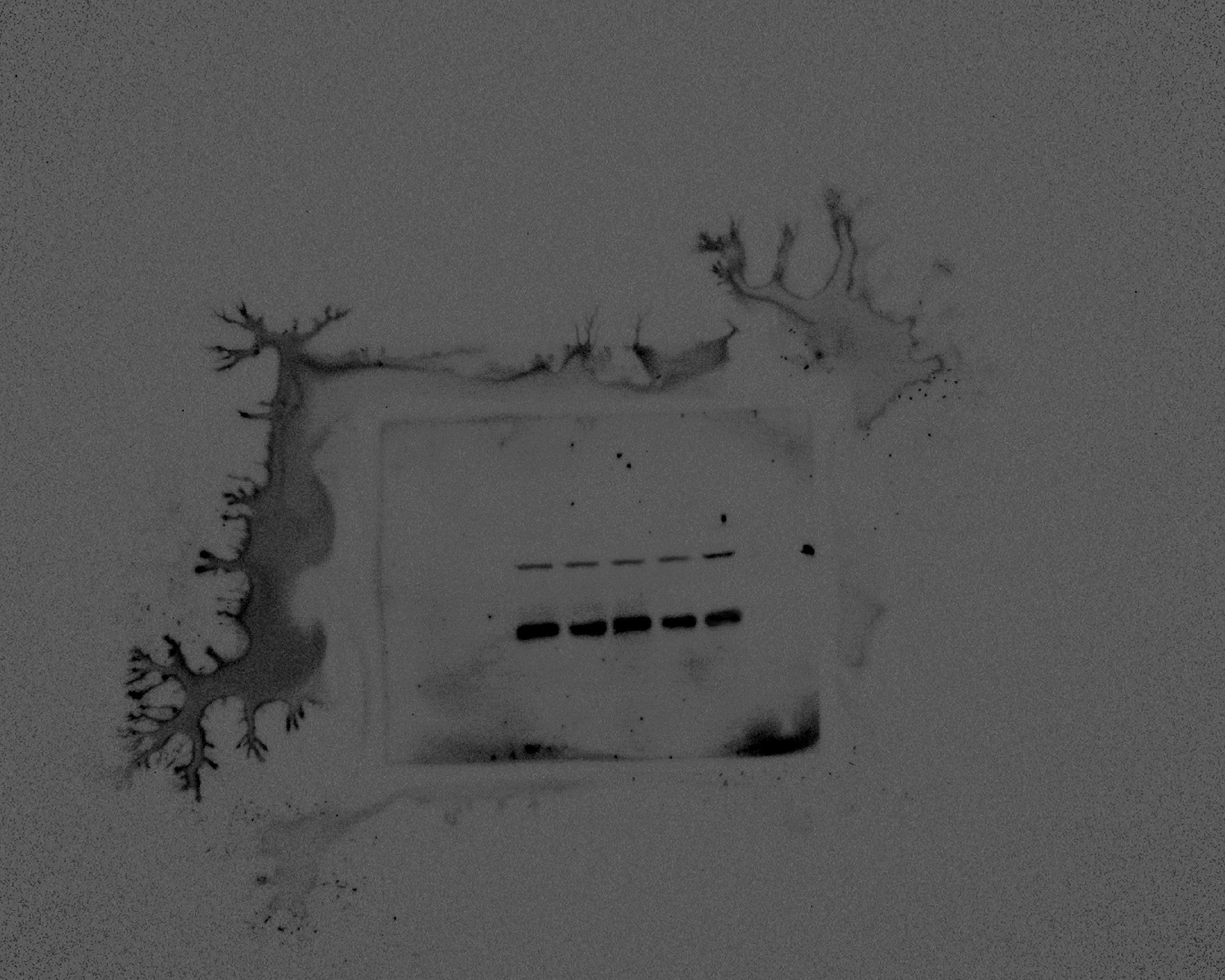

Supplement: Supplementary file 3 [file Data_Sheet_2.ZIP › The original image/p38.jpg]

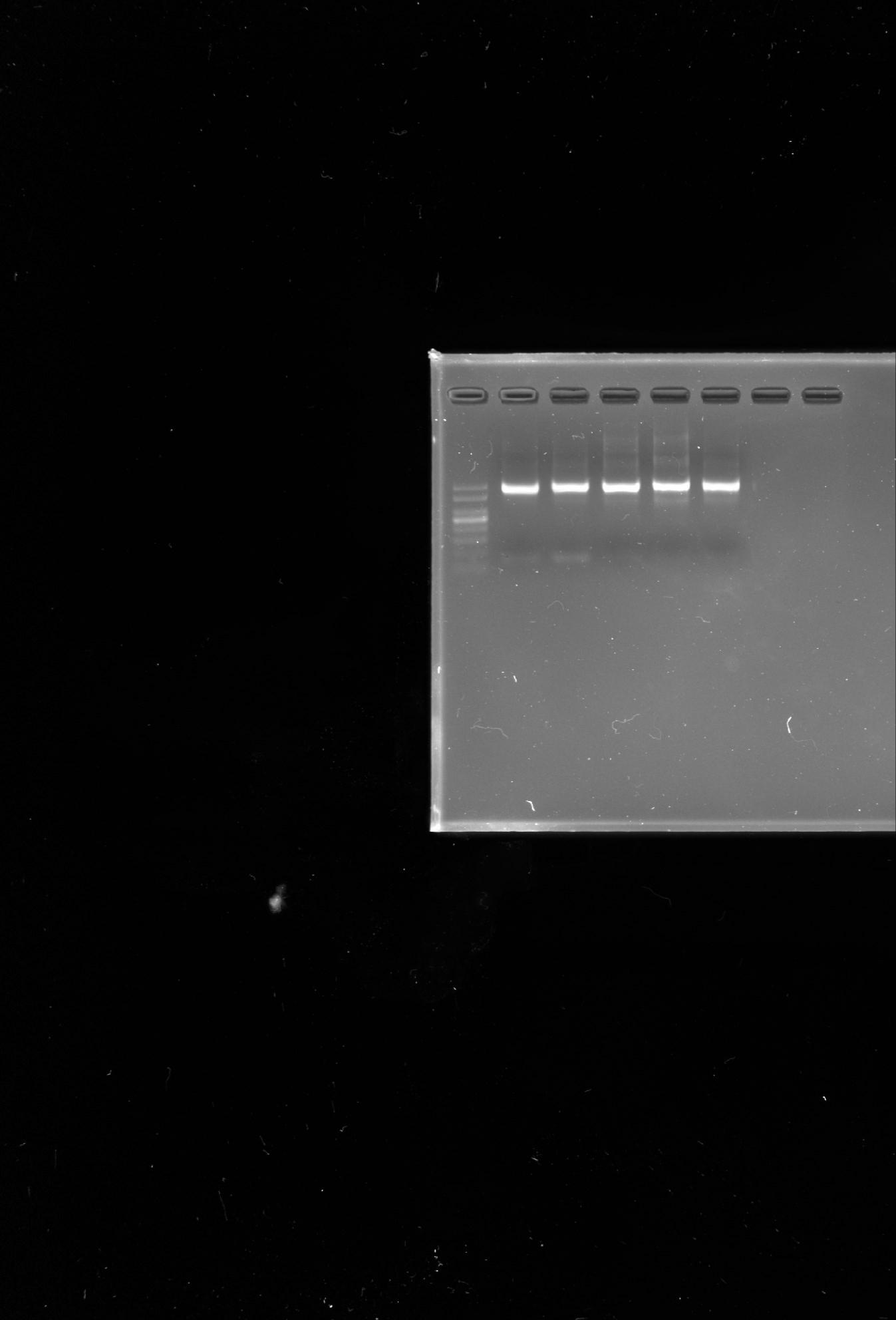

Supplement: Supplementary file 3 [file Data_Sheet_2.ZIP › The original image/pcr-GAPDH.jpg]

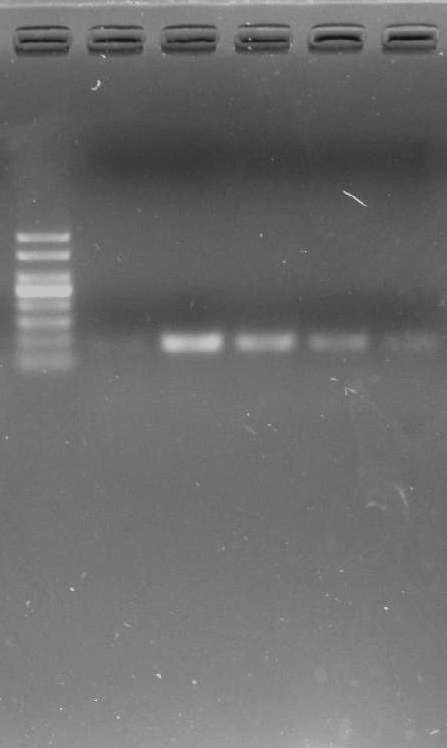

Supplement: Supplementary file 3 [file Data_Sheet_2.ZIP › The original image/TNF-α.jpg]
